# A realistic simulation-based benchmark of microbiome normalization in sample stratification and taxa-level analysis

**DOI:** 10.3389/fbinf.2026.1863340

**Published:** 2026-07-06

**Authors:** Amen Al Khafaji, Daniel Vallejo-España, Carolina Gómez-Llorente, José Camacho

**Affiliations:** 1 Research Centre for Information and Communication Technologies (CITIC-UGR), University of Granada, Granada, Spain; 2 Biomedical Engineering Department, University of Technology, Baghdad, Iraq; 3 Department of Biochemistry and Molecular Biology II, School of Pharmacy, Institute of Nutrition and Food Technology José Mataix, Biomedical Research Center, University of Granada, Granada, Spain; 4 Instituto de Investigación Biosanitaria ibs.GRANADA, Granada, Spain; 5 CIBEROBN (Physiopathology of Obesity and Nutrition CB12/03/30038), Instituto de Salud Carlos III, Madrid, Spain; 6 Spanish Network in Maternal, Neonatal, Child and Developmental Health Research (RICORS-SAMID, RD24/0013/0007), Instituto de Salud Carlos III, Madrid, Spain

**Keywords:** metagenomics, microbiome, normalization, observation-based missing data methods for exploratory data analysis, principal component analysis, sequencing depth

## Abstract

**Motivation:**

Normalization is a critical step in microbiome studies because sequencing depth and sparsity can strongly affect downstream analyses. In real datasets, however, the underlying biological signal is unknown, making it difficult to determine whether a normalization method preserves true group differences or introduces distortions. To address this problem in a way that remains relevant to real applications, we developed a simulation-based evaluation framework informed by real microbiome data. The framework generates realistic datasets with known ground truth and enables quantitative comparison of normalization methods at both the sample and taxa levels.

**Results:**

Method performance depended on taxonomic resolution and on whether sequencing depth was confounded with group structure. In our case study, model-based normalization-factor methods, particularly edgeR-TMM and, in some settings, DESeq2, gave the closest match to the simulated biological contrast, indicating better recovery of taxa-level differences while preserving sample-level separation. TSS and rarefaction were often the next-best performers. Shannon diversity analyses further showed that sequencing-depth differences alone could create false-positive group differences for several methods, whereas rarefaction remained closest to nominal Type I error control. These results also showed that visual or statistical sample separation alone was not sufficient to judge normalization performance, because apparent group differences did not always correspond to correct taxa-level recovery. Rather than identifying a universally best method, the proposed framework provides a coherent strategy for evaluating existing and new normalization approaches under realistic, data-dependent scenarios.

## 1 Introduction

Understanding human microbiome composition is essential for clarifying how specific microbes interact with the host. Alterations in microbial composition, known as dysbiosis, are commonly associated with disease states. Studying the human microbiome therefore supports the development of personalized diagnostic and therapeutic strategies (Kashyap et al., 2017; Petrosino, 2018; Wang, 2022). Metagenomic studies capture host–microbe interactions by characterizing the diversity and abundance of microbial species in a sample using high-throughput DNA sequencing (Wang, 2022). To profile microbial communities, two main sequencing approaches are commonly used: shotgun sequencing and marker gene analysis (Pérez-Cobas et al., 2020; Knight et al., 2018; Sharpton, 2014). Shotgun sequencing captures all microbial genes present in a sample, whereas marker gene analysis targets a single phylogenetically informative gene, typically 16S or 18S rRNA (Durazzi et al., 2021; Madison et al., 2023). Among marker gene approaches, 16S rRNA sequencing is widely used when the aim is to characterize the microbial composition of a sample (Durazzi et al., 2021; Lutz et al., 2022; Lin and Peddada, 2020). Following sequencing, reads are processed to generate a taxa abundance table, representing counts of each taxonomic group across samples. These taxonomic units are defined as either Operational Taxonomic Units (OTUs), obtained through clustering, or Amplicon Sequence Variants (ASVs), derived from error-correction methods (Chiarello et al., 2022; Peng and Dorman, 2021; Hong et al., 2022). The sum of counts for each sample, known as its sequencing depth or library size, varies considerably between samples (Peng and Dorman, 2021). Metagenomic datasets, like other omics data, are affected by both biological and technical sources of variation. Among these, differences in sequencing depth across samples represent a major challenge, affecting the accuracy of downstream analyses (Peng and Dorman, 2021). Normalization is therefore essential to ensure comparability between samples. Yet, there is no consensus on the best way to normalize these data. A common normalization approach is Total Sum Scaling (TSS) (Yang and Chen, 2022), in which all samples are scaled to the same sum of counts (Xia, 2023; Paulson et al., 2013). Rarefaction, also called subsampling, randomly resamples reads from each sample to a fixed sequencing depth, which may lead to the loss of rare or low-abundance species and reduce statistical power (Chiarello et al., 2022; Peng and Dorman, 2021; Paulson et al., 2013; Gloor et al., 2017; McMurdie and Holmes, 2014; Pereira et al., 2018; Willis, 2019). Beyond these approaches, count-based statistical methods originally developed for transcriptomics, such as DESeq (Calle, 2019; McGee et al., 2019; Anders and Huber, 2010) and edgeR (Calle, 2019; McGee et al., 2019; Robinson et al., 2010), provide alternatives that account for differences in sequencing depth without discarding data. Library-size normalization adjusts for differences in sequencing depth but does not account for the compositional nature of microbiome data, where counts are constrained by a fixed total. Analyses must therefore focus on relative abundances rather than absolute counts (Gloor et al., 2017). Compositional Data Analysis (CoDA) provides statistical methods designed for such data (Weiss et al., 2017; Greenacre, 2021; Filzmoser et al., 2018). A common approach is the Centered Log-Ratio (CLR) transformation, in which each taxon’s count is divided by the geometric mean of all taxa in a sample and log-transformed (Filzmoser et al., 2018; Wang and Luan, 2023). The choice of an optimal normalization method remains challenging, and recommendations in different studies are often in conflict (McMurdie and Holmes, 2014; Weiss et al., 2017; Nearing et al., 2022; Schloss, 2024; Knight et al., 2018), as shown in Table 1. Existing evaluations typically focus on a single analytical aspect, either at the sample level (stratification or clustering, often performed through Principal Coordinates Analysis (PCoA)) or at the feature level (differential abundance, using some diversity inferential test). Rarely both aspects are assessed within an integrated framework. Existing evaluations typically focus on a single analytical aspect, either on sample-level structure (stratification or clustering, often assessed through ordination) or on taxon-level contribution. This narrow focus provides only a partial view of the performance of the method. Another important limitation is that studies based on real microbiome data lack a known reference structure, making it difficult to determine whether a normalization method preserves the true biological signal or introduces distortion (Kohnert and Kreutz, 2025; Gamboa-Tuz et al., 2025). In simulation-based studies, this problem can be addressed by defining the underlying community composition of each group in advance. This known composition serves as the ground truth, because it provides an explicit reference against which the effect of normalization can be evaluated. Without such a reference, differences observed after normalization cannot be assessed in terms of accuracy, but only described qualitatively. Similarly, many simulations may not fully capture the compositional nature of microbiome data, thereby limiting the interpretability and reliability of their conclusions. In this paper, we aim to develop a framework to assess normalization methods in metagenomic data. To address previous gaps, we introduce a realistic simulation framework, starting from real measurements, which incorporates a multivariate analytical approach based on Principal Component Analysis (PCA) and observation-based Missing data methods for Exploratory Data Analysis (oMEDA) (Camacho, 2011). By embedding PCA and oMEDA within a ground-truth simulation pipeline, the framework provides a unified platform to evaluate normalization methods. Therefore, our study’s contributions are:

- Simulation-based evaluation: We devise a Monte Carlo simulation pipeline that generates datasets starting from real microbiome data and a known ground truth.
- Integrated analytical approach: We combine sample-level diversity testing with PCA and oMEDA to develop an integrated and interpretable framework for examining normalization performance. The framework evaluates sample separation through alpha-diversity analysis and PCA score patterns, and taxa-level abundance-pattern recovery by comparing oMEDA results and the ground truth.
- Assessment of normalization accuracy: By combining the simulation and analytical approaches, we compute error estimates between the predefined biological ground truth and the estimate obtained from PCA and oMEDA, yielding a quantitative measure of accuracy for each normalization method evaluated.

**TABLE 1:** Assessment approaches applied in previous studies.

Study (data type)	Focus of the study and recommendation on normalization
McMurdie and Holmes (2014) (Simulation)	Focus: Clustering and differential abundance, evaluated in separate simulations rather than within an integrated framework. Recommendation: Against rarefying and proportions; mixture-model-based approaches performed better. Impact in the field: 3,366 citations; annual rate: 281
Weiss et al. (2017) (Real data and simulation)	Focus: Clustering and differential abundance; revisits McMurdie and Holmes (2014) using real data. Recommendation: Rarefying helps with sequencing-depth variability; against proportions. Impact in the field: 2,196 citations; annual rate: 241
McKnight et al. (2019) (Real data and simulation)	Focus: Clustering accuracy. Recommendation: In favor of proportions and rarefaction; against log transformations. Impact in the field: 399 citations; annual rate: 57
Nearing et al. (2022) (Real data)	Focus: Differential abundance. Recommendation: In favor of compositional tools such as ALDEx2 and ANCOM-II; against edgeR; consensus analysis across methods is advised. Impact in the field: 982 citations; annual rate: 231
Schloss (2024) (Simulation)	Focus: Control of uneven sequencing effort in alpha- and beta-diversity analyses. Recommendation: Rarefaction is the best current method for uneven sequencing effort. Impact in the field: 178 citations; annual rate: 81

This summary highlights how the analytical focus (for example, clustering versus differential abundance) influences recommendations on normalization methods. Citation counts and annual rates were updated in April 2026 based on currently available cited-by values. Abbreviations: ANCOM, analysis of composition of microbiomes; ALDEx2, ANOVA-Like Differential Expression version 2.

We demonstrate our evaluation paradigm in a set of simulation cases to assess popular normalization methods in terms of type I errors–the generation of artifactual effects–, type II errors–the failure to recover true biological effects–, and the combined impact of both.

## 2 Methods

### 2.1 Simulation and analysis workflow

The workflow in this study evaluates normalization methods in metagenomic data analysis through a structured process.

**FIGURE 1:**
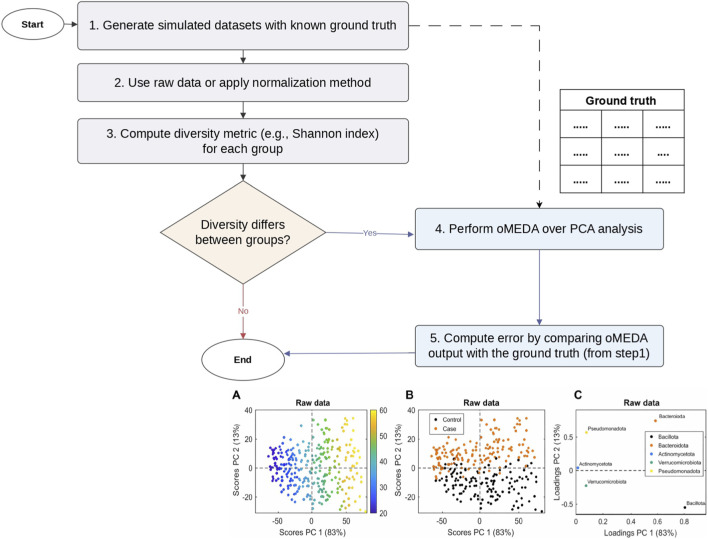
Workflow of the proposed framework for evaluating normalization methods in metagenomic data. (1) Datasets were simulated with known ground truth under different scenarios. (2) The resulting count data were analyzed either as raw data or after application of a normalization method. (3) Shannon diversity was then used as a screening step to assess whether diversity differed between groups, although it can be substituted with a different diversity metric. If a significant difference was detected, the data proceeded to (4) Principal Component Analysis (PCA) combined with observation-based Missing-data methods for Exploratory Data Analysis (oMEDA). (5) The resulting oMEDA output was then compared with the ground truth defined in Step 1 to compute the corresponding error. Scenarios without significant Shannon-diversity differences were not carried forward to downstream multivariate analysis.

It involves simulating abundance data based on a real dataset under different sequencing-depth scenarios to assess how normalization methods perform in the presence of depth-related variation, analyzing the results with PCA and oMEDA. Finally, the outputs are compared with a ground truth derived from the real dataset (Figure 1).

To illustrate the workflow, we simulate abundance data derived from real microbiome data reported by Gomez-Llorente et al. (2020). An abundance table X was generated, with rows representing individual samples and columns representing taxa (bacterial groups), and each sample labeled by condition (*control vs. case*). Simulations at two taxonomic resolutions, phylum and genus, were designed to approximate the taxonomic distribution of bacteria in the human gut microbiome while explicitly modeling technical variation. These simulations were conducted at different taxonomic levels to provide multiple scenarios, accounting for both the compositional nature and the sparsity of microbiome data. Because the composition of the simulated data, i.e., the ground truth, is known *a priori*, these datasets provide a controlled framework for quantitatively evaluating the performance of different normalization methods. By considering different sequencing depth resolutions, we were able to explore how they affect the ability of PCA and oMEDA to capture underlying biological differences. We first modeled a simulation at the phylum level, followed by a simulation at the genus level.

The simulations were intentionally performed at low sequencing depth to evaluate normalization performance under challenging conditions with stronger sampling variability and compositional effects, and an increased sparsity at the genus level. To assess the effects of normalization at depths typical of real 16S rRNA studies, we performed additional simulations at higher sequencing depth (discussed in the Supplementary Material). These two sequencing depth settings complement each other: the low-depth simulations expose potential weaknesses of normalization methods under a more extreme condition, whereas the realistic-depth simulations support the practical relevance of the findings.

#### 2.1.1 Phylum-level simulation

**FIGURE 2:**
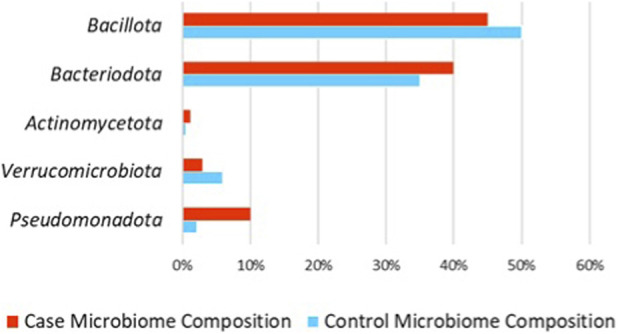
Predefined phylum-level relative abundances for the control and case groups used to generate the simulated data for the positive and unequal-depth positive scenarios.

The phylum-level simulation was constructed at the phylum taxonomic rank, where zero inflation was minimal. It comprised five taxa: *Bacillota*, *Bacteroidota*, *Actinomycetota*, *Verrucomicrobiota*, and *Pseudomonadota* (Gomez-Llorente et al., 2020). To facilitate interpretation, we modeled two groups, control and case, without introducing additional between-subject variability. Group-specific phylum probabilities were specified from the real-data-informed ground truth reported by Gomez-Llorente et al. (2020), and each sample was then generated by multinomial sampling at the target sequencing depth. We considered four simulation scenarios: (i) a positive scenario, corresponding to a differential-composition, balanced-depth setting, in which the two groups differed in composition, following the reference relative abundances shown in Figure 2, and both control and case samples were generated at depths of 100–300 reads; (ii) an unequal-depth positive scenario, corresponding to a differential-composition, imbalanced-depth setting, in which the groups differed in composition and sequencing-depth distribution, with control samples generated at 100–300 reads and case samples at 300–900 reads; (iii) a negative scenario, corresponding to a same-composition, balanced-depth setting, in which the two groups had the same composition and both control and case samples were generated at depths of 100–300 reads; and (iv) an unequal-depth negative scenario, corresponding to a same-composition, imbalanced-depth setting, in which the two groups had the same composition but differed in sequencing-depth distribution, with control samples generated at 100–300 reads and case samples at 300–900 reads. By utilizing scenarios with unequal sequencing depths in the control and case groups, we can evaluate whether normalization methods are robust to depth acting as a confounding factor. Negative scenarios allow for testing whether normalization methods introduce artifacts that might lead to false sample clustering.

We repeated the four scenarios under a more realistic setting in terms of sequencing depth. Balanced positive and balanced negative scenarios used 10,000–100,000 reads per sample in both groups, whereas unequal-depth positive and unequal-depth negative scenarios used 10,000–30,000 reads for control samples and 30,000–90,000 reads for case samples. These additional simulations assess whether the conclusions from the original low-depth setting persist at higher sequencing depths, and results are available in the Supplementary Material.

For each scenario, we generated 100 independent datasets, each containing 150 samples per group. This design isolates technical sampling variability while allowing controlled evaluation of the effects of compositional differences and sequencing-depth imbalance on downstream analysis.

#### 2.1.2 Genus-level simulation

**FIGURE 3:**
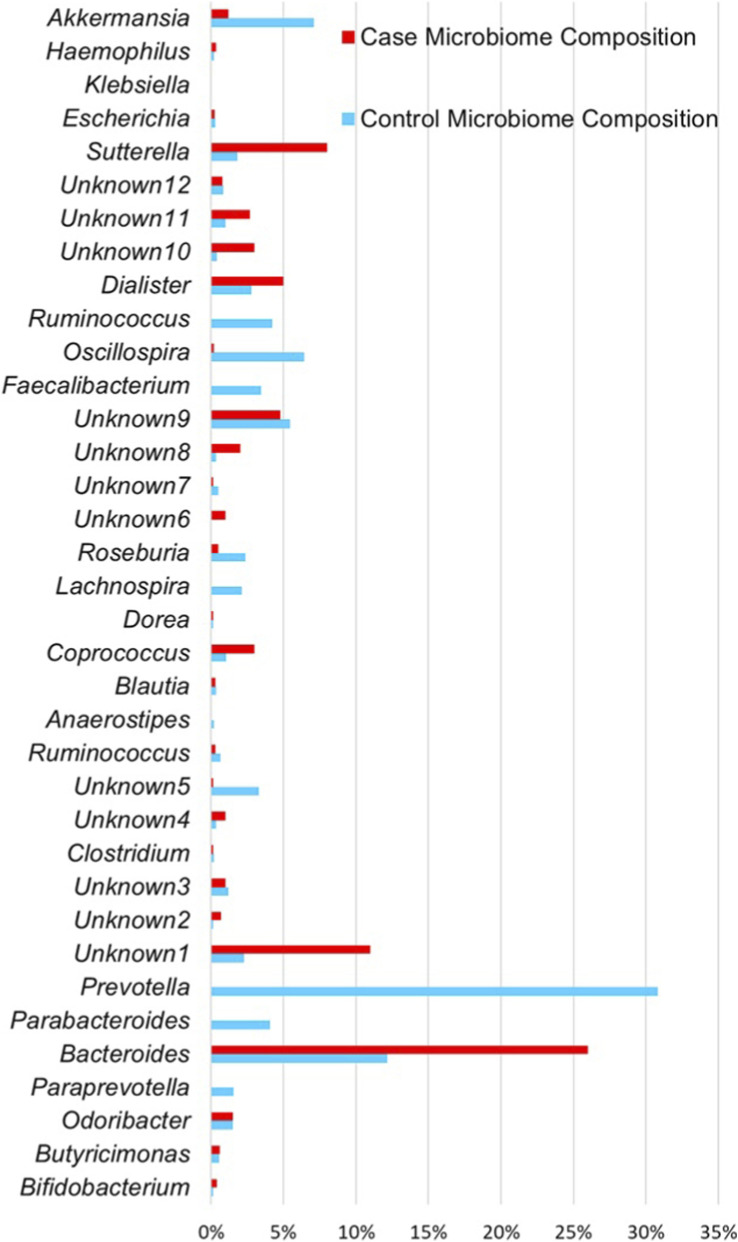
Predefined genus-level relative abundances for the control and case groups used to generate the simulated data for the positive and unequal-depth positive scenarios.

The genus-level simulation was designed to evaluate normalization methods under strong zero inflation, reflecting the higher sparsity typically observed at the genus rank. As in the phylum-level simulation, count tables were generated by first constructing a pool of one million sequences per group and then creating samples by subsampling reads with replacement. Mirroring the phylum-level simulation, the same four scenarios were considered at the genus level, with 100 independent datasets generated per scenario and 150 samples per group (control and case). In the *positive* and *unequal-depth positive* scenarios, control and case groups were generated according to the ground truth shown in Figure 3; in the latter, sequencing depth was imbalanced between groups (control: 100–300 reads; case: 300–900 reads). In the *negative* and *unequal-depth negative* scenarios, the two groups had identical genus compositions; again, the unequal-depth setting used imbalanced sequencing depth (control: 100–300 reads; case: 300–900 reads), whereas the balanced-depth setting used 100–300 reads in both groups.

This sequencing depth setting acts an informative stress-test. This setting increases sparsity and zero inflation at the genus level, particularly for rare taxa with expected abundances close to zero, thereby making the effects of sparsity, zero handling, and sequencing-depth imbalance more apparent. As for the phylum-level simulation, the four genus-level scenarios were also repeated under a more realistic setting in terms of sequencing depth, using 10,000–100,000 reads per sample for the balanced scenarios and 10,000–30,000 versus 30,000–90,000 reads for the unequal-depth scenarios. These simulations then provide a complementary check of whether the same conclusions hold under practical sequencing depths.

Genus-level microbiome matrices are often sparse, particularly when many low-abundance taxa are included, or the sampled community is highly diverse. In such settings, zero counts may arise from biological absence, limited sampling, or censoring of taxa below the detection limit, rather than representing a single uniform mechanism (Paulson et al., 2013; Chan and Li, 2024; Kumar et al., 2024). Consistent with this general property of genus-level microbiome data, the genus-level simulation in this study incorporated a sparse community structure. Specifically, the predefined ground truth contained several rare taxa with expected abundances close to zero. In the representative simulated dataset examined here, 13 taxa had expected abundances below 1% in both groups, and 60% of the corresponding count-matrix entries were zero. Thus, sparsity in this setting was both biologically motivated and an intentional feature of the genus-level simulation design.

### 2.2 Normalization approach

After creating abundance tables from the simulations, normalization methods were applied to correct for differences in sequencing depth across samples. Each method listed in Table 2 was applied to the raw abundance data. These methods include approaches that convert counts to relative abundances (e.g., TSS), estimate sample-specific scaling factors (e.g., CSS, DESeq2, and edgeR-TMM), or transform the data to a log-ratio scale (e.g., CLR).

**TABLE 2:** Normalization methods evaluated in this study.

Name	Description
None	No correction for unequal sequencing depth
Rarefaction	Subsample each sample to equal depth without replacement (Weiss et al., 2017)
Centered log-ratio (CLR)	Log-transform values relative to the geometric mean of each sample (Wang and Luan, 2023)
CLR with Bayesian-multiplicative replacement (CLR-BMR)	Applies CLR after multiplicative zero replacement (Martín-Fernández et al., 2015; Palarea-Albaladejo and Martín-Fernández, 2015)
Total sum scaling (TSS)	Divide taxa values by the sum of counts in each sample (Wang and Luan, 2023)
Cumulative sum scaling (CSS)	Scale counts using a stable quantile of each sample’s distribution, reducing the impact of high counts (Wang and Luan, 2023)
DESeq2	Computes scaling factors using geometric means and median ratios, stabilizing variance with a log-like transformation (Weiss et al., 2017)
edgeR-TMM	Computes trimmed mean of log-fold differences relative to a reference to derive scaling factors (Robinson et al., 2010; Weiss et al., 2017; Wang and Luan, 2023)
ANOVA-Like Differential Expression 2 (ALDEx2)-derived representation	Generates Monte Carlo instances from the Dirichlet distribution and applies CLR transformation (Fernandes et al., 2013; Fernandes et al., 2014)
Analysis of Compositions of Microbiomes with Bias Correction (ANCOM-BC)-derived representation	Uses bias-corrected log-abundance estimation (Lin and Peddada, 2020)

ALDEx2 and ANCOM-BC, are mainly differential-abundance methods rather than standalone normalization methods. Here, their transformed output matrices were evaluated alongside the other normalization methods.

Because CLR-based transformations are undefined in the presence of zeros, the primary CLR implementation replaced zero counts with a pseudo-count of 1 before log transformation. To examine whether the CLR results were sensitive to this zero-handling choice, we also evaluated CLR after Bayesian multiplicative replacement, reported as CLR-BMR.

### 2.3 PCA and oMEDA analysis

Multivariate methods allow the analysis and visualization of the abundance table X or data structures derived from it. In this study, we focus on PCA and oMEDA, which form the core of our pipeline. Throughout this paper, samples are arranged in the rows of X , while taxa are arranged in the columns, yielding N rows and M columns.

#### 2.3.1 Principal component analysis (PCA)

PCA is a well-known multivariate analysis method used to reduce the dimensionality of high-dimensional data while preserving most of its variance (Filzmoser et al., 2018; Sankaran and Holmes, 2019; Camacho et al., 2017). PCA generates a new representation of the data composed of orthogonal variables known as Principal Components (PCs), which capture the subspace containing maximum variance. In our pipeline, PCA is directly applied to the abundance table X after mean-centering. The PCA decomposition of matrix X is expressed in Equation 1:

$$X=T_{A}P_{A}^{T}+E_{A}$$ (1)

where $T_{A}$ is the scores matrix (N×A) , $P_{A}$ is the loadings matrix (M×A) , and $E_{A}$ is the residual matrix (N×M) .

PCA and PCoA are both ordination methods used to represent relationships among samples, but they are based on different mathematical inputs. PCA is applied directly to the abundance table X and decomposes its variance-covariance structure, whereas PCoA, also known as classical multidimensional scaling (CMDS), constructs sample coordinates from a pairwise distance or dissimilarity matrix. The two approaches are equivalent only under specific conditions: PCA on the abundance table gives the same ordination subspace as PCoA performed on the corresponding Euclidean distance matrix (Mardia et al., 1979; Gower, 1966). When PCoA is applied to non-Euclidean dissimilarities, such as Bray–Curtis or Jaccard distances, this equivalence no longer holds.

For the present framework, we chose PCA over PCoA and other dimensionality reduction approaches, such as Uniform Manifold Approximation and Projection (UMAP), because those methods do not provide a direct and interpretable link between observations and variables in the projection subspace. PCA loading matrix P provides direct taxon-level information that can be linked to scores through the oMEDA interpretation. Thus, PCA allows us to visualize group differences among samples while also identifying the taxa contributing to them.

**FIGURE 4:**
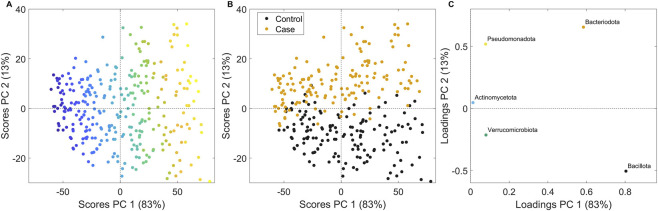
Principal Component Analysis (PCA) score and loading plots for the raw phylum-level data in the positive scenario: **(A)** score plot with samples colored by sequencing depth, **(B)** score plot with samples colored by group, and **(C)** loading plot showing the five phyla.

Figure 4 compares control and case samples using PCA on the phylum-level raw data, without normalization. In Figure 4A, the score plot shows the first two principal components. The color gradient indicates sequencing depth, which is mainly aligned with PC1, the horizontal axis, accounting for approximately 83% of the variance. Figure 4B displays the same plot colored by group, with control samples shown as black circles and case samples as orange circles. Group separation is observed primarily along PC2, the vertical axis, accounting for approximately 13% of the variance. Thus, PCA shows that the dominant source of variation in the raw count table is the library-size effect associated with sequencing depth, which is larger than the biological group difference of interest.

To quantify the relationship between sequencing depth and the PCA structure of the raw count table, we correlated the original sequencing depth of each sample with the first two principal component score vectors. Across the simulated datasets, PC1 explained approximately 83% of the variance and was strongly associated with sequencing depth, with a pearson correlation of |r|=0.987±0.001 and a spearman correlation of |ρ|=0.987±0.002 . In contrast, PC2 explained approximately 13% of the variance and showed a much weaker association with sequencing depth, with |r|=0.135±0.011 and |ρ|=0.115±0.013 . These results indicate that sequencing depth represents the dominant depth-driven axis of variation in the raw data, mainly captured by PC1 rather than PC2. When combined with the score plot, the loading plot (Figure 4C) provides information about how taxa contribute to the main patterns of variance. We may interpret that *Bacteroidota* and *Pseudomonadota* are more abundant in case samples, since loadings yield positive values in PC2, while *Bacillota* and *Verrucomicrobiota*, with negative loadings in PC2, are more abundant in control samples. Yet, the simultaneous interpretation of scores and loadings requires some experience and can become challenging in some situations, and it is also difficult to quantify. That is the reason why we incorporate oMEDA in our pipeline, as described in the next section.

**FIGURE 5:**
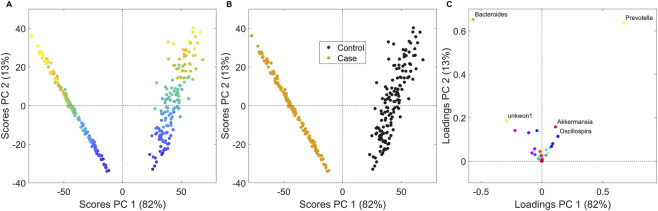
Principal Component Analysis (PCA) score and loading plots for the raw genus-level data in the positive scenario: **(A)** score plot with samples colored by sequencing depth, **(B)** score plot with samples colored by group, and **(C)** loading plot showing the 36 taxa. and the bottom panel shows the class summary plot for the same dataset.

The PCA procedure applied to the genus-level simulation with raw data is shown in Figure 5. This maintains consistency in preprocessing and visualization with the phylum-level analysis, allowing direct comparison across taxonomic resolutions. The resulting score plot indicates clear separation between control and case samples along PC1, which explained approximately 82% of the variance. In contrast to the phylum-level case, sequencing-depth differences were mainly captured by PC2, which explained approximately 13% of the variance.

To quantify this pattern, we correlated the original sequencing depth of each sample with the first two principal component score vectors. Because the sign of each principal component is arbitrary, we interpreted the strength of association using the magnitude of the correlation. PC1 showed only a weak association with sequencing depth, with a Pearson correlation of |r|=0.075±0.054 and a Spearman correlation of |ρ|=0.049±0.039 . In contrast, PC2 was strongly associated with sequencing depth, with a Pearson correlation of |r|=0.975±0.006 and a Spearman correlation of |ρ|=0.975±0.006 .

This represents a more favorable situation than the phylum-level case, because the main control–case separation is captured by PC1, while depth variation is mainly represented by PC2. Nevertheless, the genus-level analysis remains challenging due to the larger number of taxa and the increased probability of zero values.

The corresponding loading plot identifies taxa-level contributions, with *Prevotella* (at the far right of PC1) enriched in control samples and *Bacteroides* (at the far left of PC1) enriched in case, highlighting the taxa driving group differences. Supplementary raw-data PCA plots are provided for the phylum and genus level realistic-depth simulations (Supplementary Figures S9, S10).

#### 2.3.2 Observation-based missing data methods for exploratory data analysis (oMEDA)

oMEDA (Camacho, 2011) is an exploratory tool designed to facilitate the interpretation of relationships between the scores $\left(T_{A}\right)$ and loadings $\left(P_{A}\right)$ in multivariate models, such as PCA and Partial Least Squares (PLS) (Abdi, 2003; Camacho et al., 2017). It identifies variables contributing to specific patterns in the observations, including clusters, trends, or outliers. For that, the input to oMEDA is the multivariate model along with a specific numerical coding of the observations, with the coding describing the pattern of interest. In the case of this paper, we use a coding associated to the two groups, with control samples identified by code −1 and case by code 1. This coding allows us to understand the relative relevance of the loadings (i.e., the taxa) to the averaged separation between the scores of the two groups in the PCA model. The output of oMEDA is a vector with that relative relevance, where positive values identify enriched taxa for case samples and negative values for control samples. This can also be visualized as a bar plot that we call the oMEDA plot. The method is available through the MEDA toolbox (Camacho, 2019).

**FIGURE 6:**
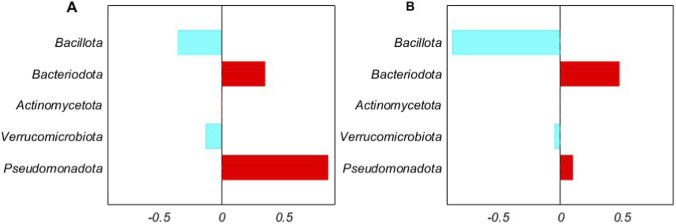
Phylum-level comparison between the oMEDA result and the corresponding ground truth for the positive scenario. **(A)** Ground-truth phylum-level contrast derived from the predefined control and case compositions using a mean-centered, squared, sign-preserving transformation (Gomez-Llorente et al., 2020). **(B)** Observation-based Missing-data methods for Exploratory Data Analysis (oMEDA) representation of the raw phylum-level data. Positive values indicate taxa associated with case samples, whereas negative values indicate taxa associated with control samples.

**FIGURE 7:**
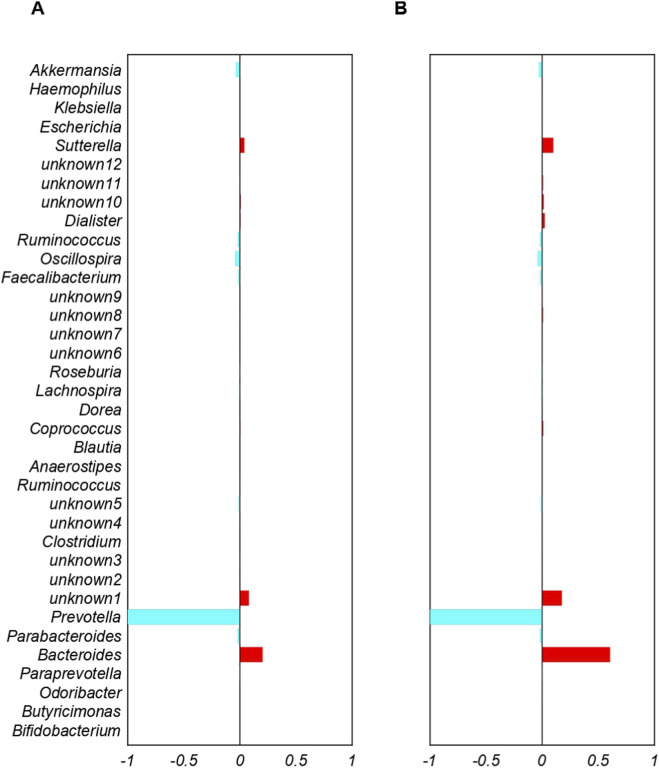
Genus-level comparison between the oMEDA result and the corresponding ground truth for the positive scenario. **(A)** Ground-truth genus-level contrast derived from the predefined control and case compositions using a mean-centered, squared, sign-preserving transformation (Gomez-Llorente et al., 2020). **(B)** Observation-based Missing-data methods for Exploratory Data Analysis (oMEDA) representation at the genus level for raw, unnormalized data in the positive scenario. Positive values indicate taxa associated with case samples, whereas negative values indicate taxa associated with control samples.

To illustrate how oMEDA simplifies the interpretation of PCA, we applied it to phylum-level and genus-level data without normalization, in order to identify taxa driving differences between control and case groups. At the phylum level, Figure 6A, the oMEDA plot highlights taxa associated with each group. The analysis on non-normalized data only allows identifying the association of *Bacillota* (with a large negative bar) to control samples (code −1 ), and of *Pseudomonadota* (with a large positive bar) to case (code 1). If we compare this result to the ground-truth in Figure 6B, we can see that oMEDA is only partially right. The mismatch between oMEDA and the ground-truth is the result of the confounding influence of the technical variability in the non-normalized data. Bearing this idea in mind, we can assess the quality of a normalization method by analyzing the resulting data with PCA, and comparing the oMEDA result with the ground-truth. This comparison combines the accuracy in the clustering of samples (in our case, how well control samples are differentiated from case samples in PCA) with the accuracy of recovery of the taxa-level abundance pattern associated with the biological effect (how well taxa associated with the biological effect are identified). The same comparison is repeated at genus level (Figure 7A vs. Figure 7B). In this case, the main taxa associated with the biological effect are mostly identified. Recall from Figure 5 that this second simulation is more favorable in terms of the amount of biological variance in comparison to the technical variance, and this is reflected in the oMEDA accuracy. Yet, there is still a lot of information missing about low-abundance differences. Normalization may improve this situation. The role of oMEDA in this paper is to facilitate the interpretation of the connection between an effect of interest in the score plot (the difference between control and case conditions) and the taxa associated with such an effect. The oMEDA vector is also a numerical structure that can be easily compared to the ground-truth, and error measures between both of them can be easily computed.

### 2.4 Performance benchmarking against the reference standard

Figures 6B, 7B respectively illustrate the ground truth for the phylum and genus simulations. Positive values indicate taxa more abundant in case samples, whereas negative values indicate taxa enriched in control samples. Since oMEDA is based on squared distances between the two conditions, we computed the ground truth from the predefined taxa proportions using squared, sign-preserving differences. This step allows a direct comparison between the oMEDA results and the ground truth. Mean-centering is also applied in the oMEDA setting; accordingly, taxa abundances were first mean-centered across the control and case conditions when constructing the ground truth Equation 2 before computing the between-group contrast Equation 3.

$${\tilde{G}}_{i,j}=G_{i,j}-\frac{G_{i,1}+G_{i,2}}{2},\;j\in \left\{1,2\right\},$$ (2)

where $G_{i,1}$ is the percentage of taxon i in control samples and $G_{i,2}$ is the percentage of taxon i in case samples.

Then, the squared (sign-preserving) ground-truth contrast was computed as:

$$gt_{i}=sign\left({\tilde{G}}_{i,2}\right)\;{\tilde{G}}_{i,2}^{2}-sign\left({\tilde{G}}_{i,1}\right)\;{\tilde{G}}_{i,1}^{2},$$ (3)

where $gt_{i}$ denotes the ground-truth difference for taxon i . Subsequently, both the ground truth vector and the corresponding oMEDA output vector were scaled to unit length to ensure comparability.

The performance of each normalization method was quantified using the error metric shown in Equation 4 defined as the squared deviation between the oMEDA output and the ground truth (both scaled to unit length);

$$e=\underset{r}{\sum }{\left(u_{r}-gt_{r}\right)}^{2},$$ (4)

where $u_{r}$ represents the oMEDA differences between groups (for any normalization approach described in Section 3), and $gt_{r}$ denotes the ground truth differences. The normalization method achieving the lowest error was considered the best fit for the simulated dataset. The same procedure was applied at the genus level.

## 3 Results and discussion

### 3.1 Alpha-diversity analysis at phylum and genus level

Alpha-diversity was evaluated under the four simulation scenarios. The aim was to assess whether the normalization methods preserved the expected group differences in Shannon diversity (or absence thereof) and whether these patterns remained stable across repeated simulations.

To quantify how effectively each normalization method preserved group-level disparities, we performed comparative statistical testing on the Shannon diversity indices for each normalized dataset (Shannon, 1948). For each dataset, we identified the “low depth” subset (the third of samples with the fewest sequences) and the “high depth” subset (the third of samples with the most sequences). Each strata contains the same amount of control samples and case samples. Evaluating alpha-diversity on the complete dataset and both depth strata independently allows us to determine if our findings remain consistent regardless of sequencing depth. Each method was assessed by calculating the frequency of significant vs. non-significant outcomes (at significance level 0.05) across 100 independently replicated datasets. This approach allowed us to evaluate the methods based on their statistical power (in the positive scenarios) and their ability to control for Type I errors (in the negative scenarios). We employed the Kruskal–Wallis test because of its capacity to generalize to datasets featuring more than the two groups (control and case) considered in this study (Kruskal and Wallis, 1952). Note that, when considering two groups, as is the case in the present study, the Kruskal–Wallis test is equivalent to the Wilcoxon rank-sum test.

At the phylum level, the Kruskal–Wallis results for Shannon diversity were consistent with the four simulated scenarios. In both the positive and unequal-depth positive scenarios, all normalization methods except ALDEx2 yielded significant results in all 100 simulation repetitions for the low-depth, high-depth, and full-dataset analyses (Table 3), indicating complete detection of the simulated diversity difference under both balanced and imbalanced sequencing-depth settings.

**TABLE 3:** Number of significant Kruskal–Wallis tests (out of 100 simulation repetitions) across normalization methods for the phylum-level Shannon diversity analysis under four simulation scenarios.

Method	Positive			Unequal-depth positive			Negative			Unequal-depth negative		
	Low	High	Full	Low	High	Full	Low	High	Full	Low	High	Full
Raw data	100	100	100	100	100	100	7	1	6	18	14	32
CLR	100	100	100	100	100	100	8	0	5	88	67	99
CLR-BMR	100	100	100	100	100	100	9	5	5	14	8	28
CSS	100	100	100	100	100	100	7	1	6	18	14	32
DESeq2	100	100	100	100	100	100	7	1	6	18	14	32
Rarefaction	100	100	100	100	100	100	5	8	8	1	10	8
TSS	100	100	100	100	100	100	7	1	6	18	14	32
edgeR-TMM	100	100	100	100	100	100	7	1	6	18	14	32
ALDEx2	87	95	100	72	92	100	8	6	7	29	35	77

Entries indicate the number of significant Kruskal–Wallis tests at α=0.05 across 100 simulated datasets. The positive and unequal-depth-positive scenarios assess detection performance, where values near 100 indicate consistent recovery of the diversity difference and lower values imply a higher Type II error risk. The negative and unequal-depth-negative scenarios assess Type I error control, where values near 5 are expected by chance alone under nominal error control. Abbreviations: CLR, centered log-ratio; CLR-BMR, CLR with Bayesian-multiplicative replacement; CSS, cumulative sum scaling; TSS, total sum scaling; ALDEx2, ANOVA-Like Differential Expression version 2; ANCOM-BC was not included in the Shannon diversity analysis because its transformed output is not a valid input for Shannon index calculation and therefore did not provide interpretable Kruskal–Wallis test results; edgeR-TMM, trimmed mean of M-values normalization implemented in edgeR; DESeq2, median-ratio size-factor normalization implemented in DESeq2.

In the negative scenario, where no biological difference was present and sequencing depth was balanced between groups, correct nominal Type I error control would correspond to a number of significant results close to the nominal significance level. This pattern was broadly observed for raw data and all normalization methods, indicating that false-positive detections remained limited under balanced null conditions.

In the unequal-depth negative scenario, where group composition was identical but sequencing depth differed between groups, values close to the nominal significance level indicate appropriate nominal Type I error control. Under this setting, rarefaction remained closest to that expectation, whereas raw data, CSS, DESeq2, TSS, ALDEx2 and edgeR-TMM showed moderate false-positive inflation and CLR showed severe inflation. This suggests that, for Shannon diversity at the phylum level, some normalization methods may introduce or amplify depth-related artifacts when group compositions are identical, rather than removing them. Inflation was generally most evident in the full-dataset analysis, indicating that pooling all samples did not necessarily stabilize inference when depth imbalance remained unresolved.

At the genus level, the Kruskal–Wallis results for Shannon diversity were also consistent with the four simulated scenarios. In both the positive and unequal-depth positive scenarios, all normalization methods, except ALDEx2 in the latest scenario, yielded significant results in at least 95 of the 100 repetitions for the low-depth, high-depth, and full-dataset analyses (Table 4), indicating accurate detection of the simulated diversity difference under both balanced and imbalanced sequencing-depth settings.

**TABLE 4:** Number of significant Kruskal–Wallis tests (out of 100 simulation repetitions) across normalization methods for the genus-level Shannon diversity analysis under four simulation scenarios.

Method	Positive			Unequal-depth positive			Negative			Unequal-depth negative		
	Low	High	Full	Low	High	Full	Low	High	Full	Low	High	Full
Raw data	100	100	100	100	100	100	0	4	4	100	92	100
CLR	100	100	100	100	100	100	6	8	6	100	100	100
CLR-BMR	100	100	100	100	100	100	3	8	7	50	21	72
CSS	100	100	100	100	100	100	0	4	4	100	92	100
DESeq2	100	100	100	100	100	100	0	4	4	100	92	100
Rarefaction	100	100	100	100	100	100	2	7	6	2	6	5
TSS	100	100	100	100	100	100	0	4	4	100	92	100
edgeR-TMM	100	100	100	100	100	100	0	4	4	100	92	100
ALDEx2	100	100	100	100	95	100	7	5	10	10	63	83
ANCOM-BC	100	100	100	81	83	87	12	9	17	75	85	91

Entries indicate the number of significant Kruskal–Wallis tests at α=0.05 across 100 simulated datasets. Under the positive and unequal-depth-positive scenarios, values near 100 indicate consistent detection of the diversity difference across repetitions, whereas lower values imply a higher Type II error risk. Under the negative and unequal-depth-negative scenarios, values near 5 indicate nominal Type I error control. Abbreviations: CLR, centered log-ratio; CLR-BMR, CLR with Bayesian-multiplicative replacement; CSS, cumulative sum scaling; TSS, total sum scaling; ALDEx2, ANOVA-Like Differential Expression version; ANCOM-BC, analysis of compositions of microbiomes with bias correction; edgeR-TMM, trimmed mean of M-values normalization implemented in edgeR; DESeq2, median-ratio size-factor normalization implemented in DESeq2.

In the negative scenario, values close to the nominal significance level (5 out of 100 significant repetitions) are observed for most methods, indicating that false-positive detections remained limited under balanced null conditions. ANCOM-BC, however, displayed a higher number of false positives.

Under the unequal-depth negative scenario, rarefaction remained closest to the expected number of false positives, whereas raw data and all other normalization methods all showed substantial inflation of significant results. This indicates that, for Shannon diversity at the genus level, sequencing-depth imbalance among control and case samples was sufficient to induce strong artifactual group differences for most methods when the underlying group compositions were identical. As for the phylum-level simulations, inflation was evident across low-depth, high-depth, and full-dataset analyses.

We also examined whether the same patterns persisted under a more realistic sequencing-depth range. These additional results, based on balanced depths of 10,000–100,000 reads and unequal-depth ranges of 10,000–30,000 versus 30,000–90,000 reads per sample, are reported in Supplementary Tables S9–S18. While the increased sequencing depth helped reduce false positives in the unequal-depth negative scenario, most methods still exhibited an inflated Type I error at the genus level. Therefore, normalization can still introduce artifacts at more realistic depth ranges.

### 3.2 Multivariate structure analysis by PCA and oMEDA

This section describes the estimated error ratios obtained for the different normalization methods across 100 simulations. The estimated error ratios were calculated for both phylum- and genus-level datasets. Both taxonomic resolutions were evaluated under two scenarios: positive and unequal-depth positive. The negative scenarios were excluded from the multivariate analysis because there are no biological differences between the control and case groups, and the corresponding score plots (Supplementary Figures S1, S2) showed substantial overlap between groups with no clear discriminative structure.

In all scenarios, the error values were compared across methods using the Analysis of Variance (ANOVA) test, considering three factors: the normalization method, the depth stratum (low-depth samples, high-depth samples and complete dataset) and the repetition (each of the 100 simulation repetitions). The ANOVA results (Supplementary Tables S19, S22) reveal that the normalization method is always a statistically significant factor, accounting for over 90% of the variance across all scenarios. This demonstrates that the choice of normalization is responsible for nearly all observed differences in estimated error, confirming its influence on the recovery of the taxa-level abundance pattern associated with the control-case contrast.

#### 3.2.1 Phylum-level simulation

Table 5 shows the estimated error ratios across normalization methods for the phylum simulation in the positive scenario. The results from *post hoc* pairwise comparisons following ANOVA are denoted by superscript letters in Table 5. All normalization methods exhibit consistent performance across the various depth groups. Notably, edgeR-TMM outperforms all the other methods. TSS, rarefaction and the preprocessing in ANCOM-BC perform similarly to raw data, and they outperform CLR, DESeq2, ALDEx2 and CSS, with the latter consistently showing the highest error rates. In the unequal-depth positive scenario, the results (Table 5) are similar to the previous case. edgeR-TMM exhibits the lowest overall error, followed by TSS, rarefaction and ANCOM-BC. However, raw data displays a significantly higher error than any normalization method, highlighting when dealing with prominent differences in sequencing depth across groups.

**TABLE 5:** Estimated error after applying normalization methods (mean ± std) for the phylum positive and unequal-depth positive scenarios.

Normalization method	Positive			Unequal-depth positive		
	Low	High	Full	Low	High	Full
Raw data	$0.255\pm 0.224^{bc}$	$0.274\pm 0.158^{b}$	$0.246\pm 0.183^{bc}$	$1.852\pm 0.037^{f}$	$1.853\pm 0.022^{g}$	$1.855\pm 0.019^{h}$
CLR	$0.356\pm 0.052^{d}$	$0.382\pm 0.045^{c}$	$0.366\pm 0.026^{c}$	$0.342\pm 0.033^{c}$	$0.378\pm 0.034^{c}$	$0.359\pm 0.018^{c}$
CLR-BMR	$0.397\pm 0.066^{cd}$	$0.418\pm 0.049^{de}$	$0.403\pm 0.033^{d}$	$0.478\pm 0.115^{d}$	$0.464\pm 0.061^{d}$	$0.460\pm 0.051^{e}$
CSS	$1.408\pm 0.046^{f}$	$1.413\pm 0.033^{g}$	$1.412\pm 0.022^{e}$	$1.433\pm 0.041^{e}$	$1.425\pm 0.027^{f}$	$1.429\pm 0.019^{g}$
DESeq2	$0.457\pm 0.169^{e}$	$0.446\pm 0.121^{e}$	$0.463\pm 0.101^{d}$	$0.350\pm 0.142^{c}$	$0.475\pm 0.121^{d}$	$0.399\pm 0.079^{d}$
Rarefaction	$0.228\pm 0.067^{b}$	$0.238\pm 0.069^{b}$	$0.230\pm 0.039^{b}$	$0.242\pm 0.078^{b}$	$0.245\pm 0.071^{b}$	$0.236\pm 0.048^{b}$
TSS	$0.232\pm 0.061^{b}$	$0.235\pm 0.040^{b}$	$0.232\pm 0.032^{b}$	$0.239\pm 0.056^{b}$	$0.240\pm 0.037^{b}$	$0.234\pm 0.030^{b}$
edgeR-TMM	${0.104\pm 0.145}^{a}$	${0.088\pm 0.142}^{a}$	${0.083\pm 0.147}^{a}$	${0.061\pm 0.090}^{a}$	${0.058\pm 0.094}^{a}$	${0.052\pm 0.093}^{a}$
ALDEx2	$0.435\pm 0.085^{de}$	$0.497\pm 0.088^{f}$	$0.459\pm 0.049^{d}$	$0.465\pm 0.083^{d}$	$0.526\pm 0.083^{e}$	$0.491\pm 0.044^{f}$
ANCOM-BC	$0.266\pm 0.013^{b}$	$0.288\pm 0.020^{c}$	$0.273\pm 0.009^{b}$	$0.252\pm 0.006^{b}$	$0.269\pm 0.012^{b}$	$0.256\pm 0.003^{b}$

Entries are mean estimated error ± standard deviation across 100 simulated datasets. Methods sharing a letter within a column are not significantly different at the 0.01 level. The lowest mean value in each column is highlighted in bold. Abbreviations: CLR, centered log-ratio; CLR-BMR, CLR with Bayesian-multiplicative replacement; CSS, cumulative sum scaling; TSS, total sum scaling; ALDEx2, ANOVA-Like Differential Expression version; ANCOM-BC, analysis of compositions of microbiomes with bias correction; edgeR-TMM, trimmed mean of M-values normalization implemented in edgeR; DESeq2, median-ratio size-factor normalization implemented in DESeq2.

**FIGURE 8:**
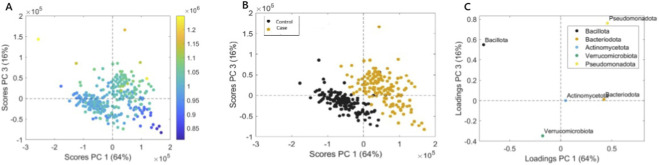
Principal Component Analysis (PCA) score and loading plots for the edgeR-TMM-normalized phylum-level data in the positive scenario: **(A)** score plot with samples colored by sequencing depth, **(B)** score plot with samples colored by group, and **(C)** loading plot showing the five phyla.

PCA visualizations for a dataset normalized with edgeR-TMM in the positive scenario are shown in Figure 8. The score plot colored by sequencing depth (Figure 8A) shows that sequencing depth is partially homogenized but not completely removed.

**FIGURE 9:**
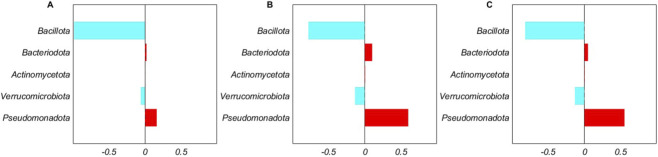
Observation-based Missing-data methods for Exploratory Data Analysis (oMEDA) representations for the phylum-level positive scenario after **(A)** edgeR-TMM normalization, **(B)** Total Sum Scaling (TSS), and **(C)** rarefaction. Blue bars indicate taxa associated with control samples, whereas red bars indicate taxa associated with case samples.

The score plot colored by condition (Figure 8B) displays a clear group separation, which mainly appears in the subspace spanned by PC1 and PC3. The loading plot (Figure 8C) reveals a relatively dispersed structure of the taxa at the phylum level. In addition to the quantitative error metrics, inspection of the PCA and oMEDA plots provides further insight into how normalization affects interpretability at the phylum level. Although PCA projections may sometimes suggest that TSS or rarefaction yield a clearer separation in the PC1–PC2 plane (Supplementary Figure S3), such visual simplicity does not necessarily imply greater fidelity to the underlying biological structure. For edgeR-TMM, part of the biological signal appears to be distributed across a higher-dimensional subspace rather than concentrated within the first two principal components, making the PCA pattern less visually direct despite its superior quantitative performance. This is reflected in the loading plot, which shows how the five taxa are arranged with respect to the dominant PCA directions and therefore which taxa underlie the main multivariate structure captured in the score plot. However, the loading plot does not directly indicate which taxa are most strongly associated with the control–case contrast itself. This is clarified by oMEDA, which identifies the taxa most strongly associated with the specific group separation encoded in the observations. In this way, the loading plot provides the multivariate background, whereas the oMEDA plot provides the contrast-specific interpretation that is most directly relevant to the present benchmark. This distinction is illustrated in Figure 9, which compares the phylum-level oMEDA patterns obtained after edgeR-TMM, rarefaction, and TSS normalization, together with the corresponding raw-data and ground-truth references (Figures 6A,B). All three normalization methods recovered the same overall biological direction, with *Pseudomonadota* and *Bacteroidota* associated with case samples and *Bacillota* associated with control samples, whereas *Verrucomicrobiota* and *Actinomycetota* contributed comparatively smaller effects. However, edgeR-TMM showed the closest agreement with the ground truth, preserving both the direction and the relative magnitude of the dominant phylum-level effects more accurately than rarefaction or TSS. By contrast, rarefaction and TSS recovered the same qualitative structure but with greater distortion in the relative taxon contributions, particularly a stronger negative weighting of *Bacillota* and a weaker recovery of *Bacteroidota*. Taken together, these comparisons show that visually appealing low-dimensional projections do not necessarily correspond to the most accurate normalization and underscore the value of simulation-based ground truth for objective method evaluation. A detailed method-by-method interpretation of the phylum-level PCA scores, loadings, and oMEDA patterns is provided in Section 3 of the Supplementary Material.

#### 3.2.2 Genus-level simulation

At the genus level, Table 6 reports the estimated error ratios for the same set of normalization methods in the positive scenario. The errors at the genus level are relatively stable across sequencing depths. DESeq2 attains the lowest total error across all depth strata while edgeR-TMM shows a higher error than DESeq2. Similarly to the phylum-level positive scenario, TSS, rarefaction and raw data are outperformed by edgeR-TMM, while CSS shows the highest error.

**TABLE 6:** Estimated error after applying normalization methods (mean ± std) for the genus-level positive and unequal-depth positive scenarios.

Normalization method	Positive			Unequal-depth positive		
	Low	High	Full	Low	High	Full
Raw data	$0.081\pm 0.033^{c}$	$0.078\pm 0.020^{c}$	$0.078\pm 0.023^{c}$	$1.363\pm 0.023^{f}$	$1.361\pm 0.014^{g}$	$1.362\pm 0.016^{h}$
CLR	$0.253\pm 0.013^{d}$	$0.343\pm 0.012^{d}$	$0.298\pm 0.008^{d}$	$0.360\pm 0.014^{c}$	$0.426\pm 0.014^{d}$	$0.392\pm 0.008^{e}$
CLR-BMR	$0.322\pm 0.023^{e}$	$0.363\pm 0.025^{e}$	$0.340\pm 0.022^{e}$	$0.364\pm 0.019^{c}$	$0.392\pm 0.014^{c}$	$0.377\pm 0.010^{d}$
CSS	$0.350\pm 0.081^{f}$	$0.378\pm 0.074^{f}$	$0.369\pm 0.046^{f}$	$1.010\pm 0.060^{e}$	$0.919\pm 0.053^{f}$	$0.964\pm 0.037^{g}$
DESeq2	${0.015\pm 0.009}^{a}$	${0.001\pm 0.003}^{a}$	${0.002\pm 0.002}^{a}$	$0.354\pm 0.070^{c}$	$0.070\pm 0.021^{b}$	$0.160\pm 0.025^{c}$
Rarefaction	$0.081\pm 0.018^{c}$	$0.079\pm 0.017^{c}$	$0.079\pm 0.010^{c}$	$0.079\pm 0.016^{b}$	$0.080\pm 0.018^{b}$	$0.078\pm 0.009^{b}$
TSS	$0.080\pm 0.016^{c}$	$0.077\pm 0.011^{c}$	$0.078\pm 0.008^{c}$	$0.078\pm 0.012^{b}$	$0.077\pm 0.008^{b}$	$0.077\pm 0.005^{b}$
edgeR-TMM	$0.032\pm 0.009^{b}$	$0.029\pm 0.013^{b}$	$0.031\pm 0.010^{b}$	${0.016\pm 0.018}^{a}$	${0.024\pm 0.050}^{a}$	${0.017\pm 0.029}^{a}$
ALDEx2	$0.449\pm 0.021^{g}$	$0.531\pm 0.023^{g}$	$0.490\pm 0.012^{g}$	$0.503\pm 0.023^{d}$	$0.583\pm 0.024^{e}$	$0.542\pm 0.013^{f}$
ANCOM-BC	$0.251\pm 0.014^{d}$	$0.340\pm 0.013^{d}$	$0.297\pm 0.009^{d}$	$0.363\pm 0.018^{c}$	$0.429\pm 0.016^{d}$	$0.394\pm 0.012^{e}$

Entries are mean estimated error ± standard deviation across 100 simulated datasets. Methods sharing a letter within a column are not significantly different at the 0.01 level. The lowest mean value in each column is highlighted in bold. Abbreviations: CLR, centered log-ratio; CLR-BMR, CLR with Bayesian-multiplicative replacement; CSS, cumulative sum scaling; TSS, total sum scaling; ALDEx2, ANOVA-Like Differential Expression version; ANCOM-BC, analysis of compositions of microbiomes with bias correction; edgeR-TMM, trimmed mean of M-values normalization implemented in edgeR; DESeq2, median-ratio size-factor normalization implemented in DESeq2.

The unequal-depth positive scenario shows similarities to the same scenario at the phylum level (Table 6). Raw data displays the highest error, above all normalized datasets. edgeR-TMM is the method with the lowest error, followed by TSS and rarefaction. DESeq2 is less effective here than in the positive scenario and exhibits the greatest variability across depth groups. While it performs comparably to rarefaction and TSS in high-depth samples, it underperforms in both the low-depth subset and the complete dataset. In comparison with the phylum-level results, the genus-level analysis showed a different and more nuanced behavior. Across both the positive and unequal-depth positive scenarios, the error remained largely stable across the low-depth subset, the high-depth subset, and the full dataset for most methods, indicating that sequencing depth had a comparatively smaller impact on signal recovery at the genus level. This pattern is consistent with the PCA of the raw data, in which the biological effect already dominated the main component structure, suggesting that depth-related variability was less influential than at the phylum level. Under these conditions, normalization did not universally improve performance. CSS and log-ratio methods like CLR and the ALDEx2 preprocessing method consistently increased the error, whereas DESeq2 showed scenario-dependent behavior, performing exceptionally well in the positive scenario but less robustly in the unequal-depth positive setting. By contrast, edgeR-TMM emerged as the most consistently effective method, yielding the lowest error across all depth settings in the unequal-depth positive scenario and performing at the top level in the positive scenario as well. TSS and rarefaction formed the next-best group, with performance close to the raw data and only marginal differences between them. Taken together, these results indicate that, unlike the phylum-level case, genus-level data were intrinsically less confounded by sequencing depth, so that most normalization methods provided limited added value. The main exception was edgeR-TMM, which delivered the most accurate and stable recovery of the underlying biological structure according to the oMEDA error criterion.

This genus-level multivariate pattern should be interpreted separately from the Shannon-based null results. Although multivariate error at the genus level was relatively stable across depth strata in the positive settings, the unequal-depth negative Shannon scenario showed that sequencing-depth imbalance alone could still induce severe false-positive diversity differences for most methods. In that setting, rarefaction was the only method that remained close to nominal Type I error control.

**FIGURE 10:**
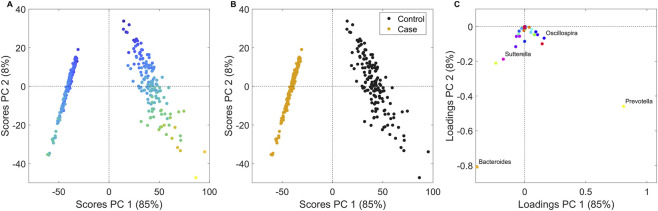
Principal Component Analysis (PCA) score and loading plots for the DESeq2-normalized genus-level data in the positive scenario: **(A)** score plot with samples colored by sequencing depth, **(B)** score plot with samples colored by group, **(C)** loading plot for the 36 genera.

**FIGURE 11:**
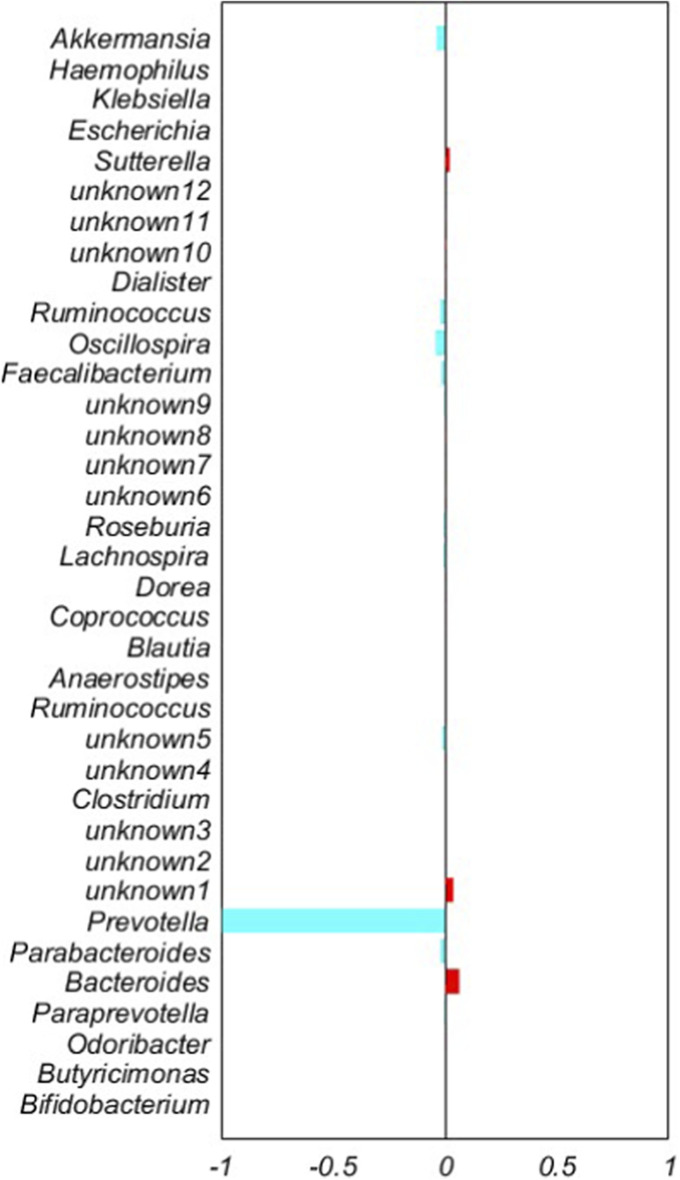
Observation-based Missing-data methods for Exploratory Data Analysis (oMEDA) representation for the genus-level positive scenario after DESeq2 normalization. Positive values indicate taxa associated with case samples, whereas negative values indicate taxa associated with control samples.

PCA of genus-level data (Figure 5B) shows a clear separation between control and case groups in the raw dataset along the first principal component, which captures 82% of the variance (Figure 5A). After DESeq2 normalization (Figure 10), the separation between groups is also clearly observed along PC1, which now captures 85% of the variance. The corresponding loading plot (Figure 10C) shows how the genera are arranged with respect to the dominant PCA directions and therefore which taxa underlie the main multivariate structure captured in the score plot. This difference is further illustrated by comparing the genus-level oMEDA plots obtained from raw data (Figure 7A), the predefined ground truth (Figure 7B), and DESeq2-normalized data (Figure 11). Whereas the loading plot provides the multivariate background by showing how taxa contribute to the dominant PCA structure, the oMEDA plot identifies the taxa most strongly associated with the specific control–case contrast and therefore gives the more direct taxa-level interpretation for the present benchmark. In the ground-truth representation, the biological signal is concentrated in a relatively small number of dominant taxa, most notably *Prevotella* and *Bacteroides*, which show strong opposing contributions between the two groups. The raw-data oMEDA plot captures this pattern only partially, with several relevant taxa appearing attenuated or less clearly separated from the background structure. After DESeq2 normalization, the oMEDA pattern more closely resembles the reference, with the dominant genera becoming more pronounced and their relative contributions more consistent with the ground truth. This comparison shows why the oMEDA representation is essential in the genus-level analysis: it makes the main taxa underlying the control–case contrast directly visible, rather than leaving them embedded within the broader multivariate structure of the PCA loadings. At the same time, the improvement after normalization is less marked than at the phylum level, which is consistent with the weaker confounding effect of sequencing depth in this genus-level positive scenario.

The evaluation of normalization methods under a higher, more realistic depth setting (Supplementary Tables S23, S24) mirrors our previous findings: the distinct behaviors and relative performance of the methods remained consistent across both positive scenarios at phylum and genus resolutions.

These results demonstrate that the impact of normalization is highly data-dependent and varies across taxonomic resolutions. While normalization is essential to mitigate sequencing-depth effects, it may be unnecessary or even detrimental when the biological signal already dominates the data structure.

## 4 Conclusion

This study shows that the effect of normalization in microbiome data cannot be judged from sample separation alone. Methods that appeared to separate groups clearly did not always recover the correct taxa-level pattern, particularly when sequencing depth was confounded with group structure. By combining simulations with known ground truth, diversity-based screening, and PCA and oMEDA-based comparison, the proposed framework makes it possible to evaluate normalization methods against an explicit biological reference rather than against visual separation alone.

Across the scenarios examined here, normalization performance depended on both taxonomic resolution and sequencing-depth imbalance. edgeR-TMM was the most consistent method overall, whereas DESeq2 performed well only in specific settings and rarefaction remained most reliable for controlling false-positive diversity differences under depth imbalance. These results argue against a universally optimal normalization strategy and instead support scenario-dependent method selection.

The main contribution of this work is therefore not the identification of a single best normalization method, but the introduction of a general benchmark for testing whether a preprocessing method preserves the biological structure of interest while limiting depth-driven distortion. While our case study utilized simulated data based on the human gut microbiome, the entire pipeline from data simulation to final analysis is applicable to other microbiome types, such as soil or oral microbiomes. We recognize that the PCA- and oMEDA-based evaluation relies on linear projections, which may not capture nonlinear relationships in microbiome data. This limitation is balanced by the interpretability of the approach, since the same linear structure allows sample-level separation and taxa-level contributions to be directly linked to the known simulated effect.

The framework can also be extended to more complex settings and used to evaluate existing or newly developed normalization approaches under controlled, biologically informed conditions. Future applications could use the same controlled simulations and normalized abundance matrices to examine whether the normalization-dependent patterns identified by PCA and oMEDA also affect downstream covariate-adjusted association analyses, including workflows based on MaAsLin2 (Mallick et al., 2021).

## Data Availability

The datasets presented in this study can be found in online repositories. The names of the repository/repositories and accession number(s) can be found below: https://github.com/AmenALkhafaji/PCA_oMEDA-for-assesst_norm_metageon.
