# Supplementary material for: A realistic simulation-based benchmark of microbiome normalization in sample stratification and taxa-level analysis
Source: Front Bioinform. 2026 Jul 6;6:1863340. doi: 10.3389/fbinf.2026.1863340 (PMC13381433; doi:10.3389/fbinf.2026.1863340)
Supplement: Supplementary file 1 [file DataSheet1.PDF]

Supplementary Materials: A Realistic Simulation-Based Benchmark of Microbiome Normalization in Sample Stratification and Taxa-Level Analysis Analysis

1 Alpha-diversity analysis across scenarios

This section summarizes Shannon diversity analyses for the original low-depth simulations and the additional realistic-depth simulations.

1.1 Baseline-depth simulations

The low-depth simulations correspond to the sequencing-depth design in the study, in which read counts were intentionally restricted to low ranges to stress-test preprocessing behaviour under sparse and sampling-limited conditions. These analyses provide the baseline results of the framework and are retained for comparison with the additional realistic-depth simulations. However, because these depths are lower than those typically obtained in modern 16S rRNA amplicon studies, the low-depth results should be interpreted as a controlled sensitivity setting rather than as a direct representation of standard sequencing practice.

1.1.1 Phylum-level simulation

In the positive scenario, all normalization methods showed higher Shannon diversity in the case group than in the control group across low-depth samples, high-depth samples, and the full dataset (Table S1). This pattern was consistent across the 100 replicates, with small standard deviations indicating good stability. Although the absolute Shannon values differed slightly between methods, the direction of the group difference remained the same. CLR produced generally higher Shannon values, whereas rarefaction produced slightly lower values. However, these differences mainly affected the scale of the index and not the biological interpretation. The similarity of the results obtained from raw data, CSS, DESeq2, TSS, and edgeR-TMM suggests that the phylum-level signal was strong and robust to normalization choice.

In the negative scenario, Shannon diversity was very similar between the control and

case groups across all normalization methods and sequencing depth setting (Table S2). This indicates that, when no true biological difference was present and sequencing depth remained consistent across groups, the methods did not generate an artificial separation. As in the positive scenario, CLR gave slightly higher Shannon values and rarefaction slightly lower values, but these shifts did not change the overall absence of group differences. The small standard deviations again indicate stable and reproducible results.

In the unequal depth positive scenario, the primary findings from the positive scenario were maintained: the case group showed higher diversity regardless of the method or depth strata (Table S3). The biological signal remained detectable despite unequal group sizes, and the low variability across replicates confirmed that results stayed stable under this design. As previously observed, the choice of normalization primarily affected the magnitude of the index rather than the direction of the effect.

In the unequal depth negative scenario (Table S4), diversity differed between groups for most methods, particularly CLR, and with the exception of rarefaction. Because these samples should be biologically identical, this suggests that most normalization methods can introduce artificial separation when sequencing depth acts as a confounding factor.

Table S1: Shannon diversity index across preprocessing methods for the phylum positive scenario.

	Low-depth samples		High-depth samples		Full dataset	
**Method**	**Control**	**Case**	**Control**	**Case**	**Control**	**Case**
Raw data	0.980 ± 0.008	1.098 ± 0.009	0.987 ± 0.007	1.105 ± 0.006	0.984 ± 0.004	1.102 ± 0.004
CLR	1.042 ± 0.007	1.146 ± 0.008	1.018 ± 0.006	1.129 ± 0.006	1.029 ± 0.004	1.137 ± 0.004
CLR-BMR	0.985 ± 0.009	1.100 ± 0.009	0.991 ± 0.007	1.106 ± 0.006	0.988 ± 0.005	1.103 ± 0.004
CSS	0.980 ± 0.008	1.098 ± 0.009	0.987 ± 0.007	1.105 ± 0.006	0.984 ± 0.004	1.102 ± 0.004
DESeq2	0.980 ± 0.008	1.098 ± 0.009	0.987 ± 0.007	1.105 ± 0.006	0.984 ± 0.004	1.102 ± 0.004
Rarefaction	0.975 ± 0.010	1.092 ± 0.010	0.975 ± 0.011	1.091 ± 0.011	0.975 ± 0.005	1.092 ± 0.006
TSS	0.980 ± 0.008	1.098 ± 0.009	0.987 ± 0.007	1.105 ± 0.006	0.984 ± 0.004	1.102 ± 0.004
edgeR-TMM	0.980 ± 0.008	1.098 ± 0.009	0.987 ± 0.007	1.105 ± 0.006	0.984 ± 0.004	1.102 ± 0.004
ALDEx2	0.778 ± 0.015	0.841 ± 0.019	0.762 ± 0.015	0.819 ± 0.016	0.770 ± 0.009	0.830 ± 0.010

Values are presented as mean ± standard deviation across 100 simulated datasets. ANCOM-BC was excluded because the exported phylum-level ANCOM-BC-related matrix was constant and therefore not valid for Shannon diversity testing.

Table S2: Shannon diversity index across preprocessing methods for the phylum negative simulation.

	Low-depth samples		High-depth samples		Full dataset	
**Method**	**Control**	**Case**	**Control**	**Case**	**Control**	**Case**
Raw data	0.980 ± 0.009	0.978 ± 0.009	0.987 ± 0.006	0.987 ± 0.006	0.983 ± 0.004	0.983 ± 0.004
CLR	1.042 ± 0.008	1.040 ± 0.008	1.018 ± 0.006	1.018 ± 0.006	1.028 ± 0.004	1.028 ± 0.004
CLR-BMR	0.987 ± 0.008	0.986 ± 0.010	0.990 ± 0.006	0.991 ± 0.006	0.988 ± 0.004	0.989 ± 0.005
CSS	0.980 ± 0.009	0.978 ± 0.009	0.987 ± 0.006	0.987 ± 0.006	0.983 ± 0.004	0.983 ± 0.004
DESeq2	0.980 ± 0.009	0.978 ± 0.009	0.987 ± 0.006	0.987 ± 0.006	0.983 ± 0.004	0.983 ± 0.004
Rarefaction	0.974 ± 0.010	0.973 ± 0.010	0.973 ± 0.011	0.974 ± 0.010	0.973 ± 0.006	0.974 ± 0.007
TSS	0.980 ± 0.009	0.978 ± 0.009	0.987 ± 0.006	0.987 ± 0.006	0.983 ± 0.004	0.983 ± 0.004
edgeR-TMM	0.980 ± 0.009	0.978 ± 0.009	0.987 ± 0.006	0.987 ± 0.006	0.983 ± 0.004	0.983 ± 0.004
ALDEx2	0.777 ± 0.016	0.777 ± 0.016	0.759 ± 0.014	0.761 ± 0.014	0.768 ± 0.009	0.769 ± 0.008

Values are presented as mean ± standard deviation across 100 simulated datasets. ANCOM-BC was excluded because the exported phylum-level ANCOM-BC-related matrix was constant and therefore not valid for Shannon diversity testing.

Table S3: Shannon diversity index across preprocessing methods for the phylum unequal-depth positive scenario.

	Low-depth samples		High-depth samples		Full dataset	
**Method**	**Control**	**Case**	**Control**	**Case**	**Control**	**Case**
Raw data	0.978 ± 0.010	1.109 ± 0.006	0.985 ± 0.006	1.111 ± 0.004	0.983 ± 0.005	1.110 ± 0.003
CLR	1.040 ± 0.009	1.125 ± 0.005	1.017 ± 0.006	1.119 ± 0.003	1.028 ± 0.004	1.122 ± 0.003
CLR-BMR	0.984 ± 0.009	1.108 ± 0.005	0.989 ± 0.006	1.111 ± 0.004	0.987 ± 0.004	1.110 ± 0.003
CSS	0.978 ± 0.010	1.109 ± 0.006	0.985 ± 0.006	1.111 ± 0.004	0.983 ± 0.005	1.110 ± 0.003
DESeq2	0.978 ± 0.010	1.109 ± 0.006	0.985 ± 0.006	1.111 ± 0.004	0.983 ± 0.005	1.110 ± 0.003
Rarefaction	0.973 ± 0.012	1.093 ± 0.011	0.973 ± 0.011	1.092 ± 0.010	0.974 ± 0.006	1.093 ± 0.006
TSS	0.978 ± 0.010	1.109 ± 0.006	0.985 ± 0.006	1.111 ± 0.004	0.983 ± 0.005	1.110 ± 0.003
edgeR-TMM	0.978 ± 0.010	1.109 ± 0.006	0.985 ± 0.006	1.111 ± 0.004	0.983 ± 0.005	1.110 ± 0.003
ALDEx2	0.778 ± 0.015	0.814 ± 0.014	0.763 ± 0.014	0.807 ± 0.011	0.769 ± 0.008	0.810 ± 0.008

Values are presented as mean ± standard deviation across 100 simulated datasets. ANCOM-BC was excluded because the exported phylum-level ANCOM-BC-related matrix was constant and therefore not valid for Shannon diversity testing.

Table S4: Shannon diversity index across preprocessing methods for the phylum unequal-depth negative scenario.

	Low-depth samples		High-depth samples		Full dataset	
**Method**	**Control**	**Case**	**Control**	**Case**	**Control**	**Case**
Raw data	0.977 ± 0.008	0.990 ± 0.005	0.987 ± 0.007	0.992 ± 0.004	0.983 ± 0.004	0.991 ± 0.003
CLR	1.039 ± 0.007	1.011 ± 0.005	1.019 ± 0.007	1.003 ± 0.004	1.028 ± 0.004	1.007 ± 0.002
CLR-BMR	0.981 ± 0.009	0.989 ± 0.005	0.988 ± 0.006	0.992 ± 0.004	0.985 ± 0.005	0.991 ± 0.003
CSS	0.977 ± 0.008	0.990 ± 0.005	0.987 ± 0.007	0.992 ± 0.004	0.983 ± 0.004	0.991 ± 0.003
DESeq2	0.977 ± 0.008	0.990 ± 0.005	0.987 ± 0.007	0.992 ± 0.004	0.983 ± 0.004	0.991 ± 0.003
Rarefaction	0.973 ± 0.009	0.976 ± 0.010	0.975 ± 0.011	0.972 ± 0.011	0.974 ± 0.006	0.974 ± 0.006
TSS	0.977 ± 0.008	0.990 ± 0.005	0.987 ± 0.007	0.992 ± 0.004	0.983 ± 0.004	0.991 ± 0.003
edgeR-TMM	0.977 ± 0.008	0.990 ± 0.005	0.987 ± 0.007	0.992 ± 0.004	0.983 ± 0.004	0.991 ± 0.003
ALDEx2	0.780 ± 0.016	0.746 ± 0.013	0.760 ± 0.013	0.725 ± 0.008	0.770 ± 0.008	0.735 ± 0.006

Values are presented as mean ± standard deviation across 100 simulated datasets. ANCOM-BC was excluded because the exported phylum-level ANCOM-BC-related matrix was constant and therefore not valid for Shannon diversity testing.

1.1.2 Genus-level simulation

At the genus level, the results mirrored those observed at the phylum level, though the direction of the effect was reversed in the positive scenarios, with control samples exhibiting higher diversity than case samples.

In the positive scenario, all normalization methods consistently showed higher Shannon diversity in the control group across all depth strata (Table S5). These findings remained stable across 100 replicates. While the choice of method again influenced the absolute scale of the index, it did not alter the biological interpretation. The uniformity across raw and normalized data (CSS, DESeq2, TSS, and edgeR-TMM) confirms that the genus-level signal is similarly robust.

For the negative scenario, diversity values were nearly identical between groups, confirming that no artificial separation was introduced when sequencing depth was balanced (Table S6). As previously noted, the scaling shifts of specific methods did not interfere with the overall absence of group differences.

In the unequal depth positive scenario, the biological signal remained clearly detectable (Table S7). Despite the unbalanced design, the control group maintained higher diversity across all methods, with minimal variability across replicates.

Finally, the unequal depth negative scenario (Table S8) repeated the trend seen previously: most methods, especially CLR, introduced an artificial separation between groups. This confirms that when sequencing depth varies across groups, only rarefaction appears to avoid generating these spurious differences.

Table S5: Shannon diversity index across preprocessing methods for the genus positive scenario.

	Low-depth samples		High-depth samples		Full dataset	
**Method**	**Control**	**Case**	**Control**	**Case**	**Control**	**Case**
Raw data	2.504 ± 0.016	2.177 ± 0.017	2.563 ± 0.011	2.227 ± 0.011	2.537 ± 0.008	2.204 ± 0.008
CLR	2.934 ± 0.012	2.735 ± 0.016	2.808 ± 0.009	2.555 ± 0.009	2.865 ± 0.006	2.637 ± 0.008
CLR-BMR	2.431 ± 0.008	2.316 ± 0.010	2.433 ± 0.007	2.312 ± 0.006	2.433 ± 0.004	2.314 ± 0.006
CSS	2.504 ± 0.016	2.177 ± 0.017	2.563 ± 0.011	2.227 ± 0.011	2.537 ± 0.008	2.204 ± 0.008
DESeq2	2.504 ± 0.016	2.177 ± 0.017	2.563 ± 0.011	2.227 ± 0.011	2.537 ± 0.008	2.204 ± 0.008
Rarefaction	2.469 ± 0.018	2.148 ± 0.020	2.468 ± 0.018	2.149 ± 0.016	2.469 ± 0.011	2.149 ± 0.011
TSS	2.504 ± 0.016	2.177 ± 0.017	2.563 ± 0.011	2.227 ± 0.011	2.537 ± 0.008	2.204 ± 0.008
edgeR-TMM	2.504 ± 0.016	2.177 ± 0.017	2.563 ± 0.011	2.227 ± 0.011	2.537 ± 0.008	2.204 ± 0.008
ALDEx2	2.752 ± 0.009	2.686 ± 0.008	2.752 ± 0.010	2.687 ± 0.009	2.753 ± 0.005	2.682 ± 0.004
ANCOM-BC	2.713 ± 0.079	2.437 ± 0.087	2.745 ± 0.055	2.510 ± 0.073	2.729 ± 0.062	2.475 ± 0.076

Values are presented as mean ± standard deviation across 100 simulated datasets.

Table S6: Shannon diversity index across preprocessing methods for the genus negative scenario.

	Low-depth samples		High-depth samples		Full dataset	
**Method**	**Control**	**Case**	**Control**	**Case**	**Control**	**Case**
Raw data	2.505 ± 0.014	2.502 ± 0.016	2.563 ± 0.011	2.564 ± 0.011	2.537 ± 0.008	2.536 ± 0.008
CLR	2.933 ± 0.012	2.934 ± 0.013	2.807 ± 0.009	2.809 ± 0.009	2.865 ± 0.007	2.866 ± 0.008
CLR-BMR	2.432 ± 0.008	2.432 ± 0.008	2.434 ± 0.006	2.433 ± 0.007	2.434 ± 0.004	2.433 ± 0.005
CSS	2.505 ± 0.014	2.502 ± 0.016	2.563 ± 0.011	2.564 ± 0.011	2.537 ± 0.008	2.536 ± 0.008
DESeq2	2.505 ± 0.014	2.502 ± 0.016	2.563 ± 0.011	2.564 ± 0.011	2.537 ± 0.008	2.536 ± 0.008
Rarefaction	2.471 ± 0.016	2.468 ± 0.017	2.468 ± 0.018	2.466 ± 0.017	2.469 ± 0.010	2.468 ± 0.010
TSS	2.505 ± 0.014	2.502 ± 0.016	2.563 ± 0.011	2.564 ± 0.011	2.537 ± 0.008	2.536 ± 0.008
edgeR-TMM	2.505 ± 0.014	2.502 ± 0.016	2.563 ± 0.011	2.564 ± 0.011	2.537 ± 0.008	2.536 ± 0.008
ALDEx2	2.750 ± 0.008	2.753 ± 0.009	2.752 ± 0.010	2.751 ± 0.009	2.752 ± 0.005	2.753 ± 0.005
ANCOM-BC	2.695 ± 0.074	2.699 ± 0.074	2.733 ± 0.052	2.733 ± 0.054	2.715 ± 0.059	2.717 ± 0.060

Values are presented as mean ± standard deviation across 100 simulated datasets.

Table S7: Shannon diversity index across preprocessing methods for the genus unequal-depth positive scenario.

	Low-depth samples		High-depth samples		Full dataset	
**Method**	**Control**	**Case**	**Control**	**Case**	**Control**	**Case**
Raw data	2.501 ± 0.015	2.244 ± 0.009	2.565 ± 0.012	2.261 ± 0.006	2.537 ± 0.007	2.253 ± 0.005
CLR	2.932 ± 0.011	2.482 ± 0.009	2.809 ± 0.010	2.391 ± 0.006	2.865 ± 0.007	2.431 ± 0.005
CLR-BMR	2.432 ± 0.008	2.432 ± 0.008	2.434 ± 0.006	2.433 ± 0.007	2.434 ± 0.004	2.433 ± 0.005
CSS	2.501 ± 0.015	2.244 ± 0.009	2.565 ± 0.012	2.261 ± 0.006	2.537 ± 0.007	2.253 ± 0.005
DESeq2	2.501 ± 0.015	2.244 ± 0.009	2.565 ± 0.012	2.261 ± 0.006	2.537 ± 0.007	2.253 ± 0.005
Rarefaction	2.469 ± 0.017	2.151 ± 0.019	2.470 ± 0.018	2.151 ± 0.017	2.469 ± 0.010	2.150 ± 0.012
TSS	2.501 ± 0.015	2.244 ± 0.009	2.565 ± 0.012	2.261 ± 0.006	2.537 ± 0.007	2.253 ± 0.005
edgeR-TMM	2.501 ± 0.015	2.244 ± 0.009	2.565 ± 0.012	2.261 ± 0.006	2.537 ± 0.007	2.253 ± 0.005
ALDEx2	2.750 ± 0.008	2.753 ± 0.009	2.752 ± 0.010	2.751 ± 0.009	2.752 ± 0.005	2.753 ± 0.005
ANCOM-BC	2.695 ± 0.074	2.699 ± 0.074	2.733 ± 0.052	2.733 ± 0.054	2.715 ± 0.059	2.717 ± 0.060

Values are presented as mean ± standard deviation across 100 simulated datasets.

Table S8: Shannon diversity index across preprocessing methods for the genus unequal-depth negative scenario.

	Low-depth samples		High-depth samples		Full dataset	
**Method**	**Control**	**Case**	**Control**	**Case**	**Control**	**Case**
Raw data	2.502 ± 0.015	2.585 ± 0.008	2.564 ± 0.011	2.609 ± 0.007	2.536 ± 0.007	2.599 ± 0.005
CLR	2.933 ± 0.012	2.758 ± 0.008	2.809 ± 0.009	2.699 ± 0.006	2.865 ± 0.007	2.726 ± 0.005
CLR-BMR	2.375 ± 0.009	2.398 ± 0.006	2.394 ± 0.007	2.403 ± 0.005	2.385 ± 0.004	2.400 ± 0.003
CSS	2.502 ± 0.015	2.585 ± 0.008	2.564 ± 0.011	2.609 ± 0.007	2.536 ± 0.007	2.599 ± 0.005
DESeq2	2.502 ± 0.015	2.585 ± 0.008	2.564 ± 0.011	2.609 ± 0.007	2.536 ± 0.007	2.599 ± 0.005
Rarefaction	2.468 ± 0.017	2.469 ± 0.017	2.472 ± 0.017	2.471 ± 0.019	2.470 ± 0.011	2.469 ± 0.010
TSS	2.502 ± 0.015	2.585 ± 0.008	2.564 ± 0.011	2.609 ± 0.007	2.536 ± 0.007	2.599 ± 0.005
edgeR-TMM	2.502 ± 0.015	2.585 ± 0.008	2.564 ± 0.011	2.609 ± 0.007	2.536 ± 0.007	2.599 ± 0.005
ALDEx2	2.755 ± 0.008	2.746 ± 0.009	2.752 ± 0.009	2.724 ± 0.008	2.754 ± 0.005	2.735 ± 0.005
ANCOM-BC	2.699 ± 0.071	2.743 ± 0.048	2.734 ± 0.050	2.771 ± 0.042	2.718 ± 0.056	2.759 ± 0.044

Values are presented as mean ± standard deviation across 100 simulated datasets.

1.2 Realistic-depth simulations

This section summarizes the Shannon diversity analysis used as an initial screening step before multivariate interpretation. The analysis was repeated under a realistic-depth setting, with sequencing depths ranging from 10,000 to 100,000 reads per sample. For

consistency with the original workflow, each replicate was evaluated using low-depth, high-depth, and full-dataset subsets; here, low and high denote relative strata within the realistic-depth range.

1.2.1 Phylum-level simulation

At phylum level, the positive and unequal-depth-positive scenarios showed a stable case–control Shannon diversity difference across preprocessing methods and depth strata (Tables S9 and S11). In the negative scenarios, Shannon diversity remained nearly identical between groups, and the Kruskal–Wallis counts stayed close to the nominal expectation for most methods (Tables S10, S12, and S13). Thus, at this resolution, the realistic-depth results indicate that the alpha-diversity conclusions are not driven by the originally low sequencing depths. CLR, and CLR-BMR behaved similarly, consistent with the low sparsity of the phylum table; the ANCOM-BC-derived phylum output was constant and is therefore not interpretable for Shannon diversity.

Table S9: Shannon diversity index across preprocessing methods for the phylum-level realistic-depth positive scenario.

	Low-depth stratum		High-depth stratum		Full dataset	
**Method**	**Control**	**Case**	**Control**	**Case**	**Control**	**Case**
Raw data	0.9953 ± 0.0007	1.1145 ± 0.0007	0.9951 ± 0.0003	1.1139 ± 0.0003	0.9952 ± 0.0003	1.1141 ± 0.0003
TSS	0.9948 ± 0.0007	1.1136 ± 0.0007	0.9950 ± 0.0003	1.1136 ± 0.0003	0.9949 ± 0.0003	1.1136 ± 0.0003
CLR	0.9952 ± 0.0007	1.1139 ± 0.0007	0.9951 ± 0.0003	1.1137 ± 0.0003	0.9951 ± 0.0003	1.1138 ± 0.0003
CLR-BMR	0.9948 ± 0.0007	1.1136 ± 0.0007	0.9950 ± 0.0003	1.1136 ± 0.0003	0.9949 ± 0.0003	1.1136 ± 0.0003
CSS	0.9948 ± 0.0007	1.1136 ± 0.0007	0.9950 ± 0.0003	1.1136 ± 0.0003	0.9949 ± 0.0003	1.1136 ± 0.0003
DESeq2	0.9948 ± 0.0007	1.1136 ± 0.0007	0.9950 ± 0.0003	1.1136 ± 0.0003	0.9949 ± 0.0003	1.1136 ± 0.0003
edgeR-TMM	0.9948 ± 0.0007	1.1136 ± 0.0007	0.9950 ± 0.0003	1.1136 ± 0.0003	0.9949 ± 0.0003	1.1136 ± 0.0003
Rarefaction	0.9948 ± 0.0009	1.1134 ± 0.0009	0.9948 ± 0.0010	1.1135 ± 0.0009	0.9948 ± 0.0005	1.1135 ± 0.0005
ALDEx2	0.6887 ± 0.0004	0.8053 ± 0.0027	0.6886 ± 0.0000	0.8060 ± 0.0015	0.6886 ± 0.0001	0.8058 ± 0.0012

Values are mean ± standard deviation across 100 simulated datasets. Low and high denote relative strata within the realistic-depth range. ANCOM-BC was excluded from this table because the exported phylum-level ANCOM-BC-related matrix was constant and therefore not valid for Shannon diversity testing.

Table S10: Shannon diversity index across preprocessing methods for the phylum-level realistic-depth negative scenario.

	Low-depth stratum		High-depth stratum		Full dataset	
**Method**	**Control**	**Case**	**Control**	**Case**	**Control**	**Case**
Raw data	0.9955 ± 0.0007	0.9957 ± 0.0007	0.9951 ± 0.0003	0.9952 ± 0.0004	0.9953 ± 0.0003	0.9954 ± 0.0003
TSS	0.9950 ± 0.0007	0.9949 ± 0.0007	0.9950 ± 0.0003	0.9950 ± 0.0004	0.9950 ± 0.0003	0.9949 ± 0.0003
CLR	0.9954 ± 0.0007	0.9953 ± 0.0007	0.9951 ± 0.0003	0.9951 ± 0.0004	0.9952 ± 0.0003	0.9951 ± 0.0003
CLR-BMR	0.9950 ± 0.0007	0.9949 ± 0.0007	0.9950 ± 0.0003	0.9950 ± 0.0004	0.9950 ± 0.0003	0.9949 ± 0.0003
CSS	0.9950 ± 0.0007	0.9949 ± 0.0007	0.9950 ± 0.0003	0.9950 ± 0.0004	0.9950 ± 0.0003	0.9949 ± 0.0003
DESeq2	0.9950 ± 0.0007	0.9949 ± 0.0007	0.9950 ± 0.0003	0.9950 ± 0.0004	0.9950 ± 0.0003	0.9949 ± 0.0003
edgeR-TMM	0.9950 ± 0.0007	0.9949 ± 0.0007	0.9950 ± 0.0003	0.9950 ± 0.0004	0.9950 ± 0.0003	0.9949 ± 0.0003
Rarefaction	0.9949 ± 0.0009	0.9947 ± 0.0011	0.9948 ± 0.0010	0.9948 ± 0.0010	0.9948 ± 0.0006	0.9947 ± 0.0006
ALDEx2	0.6887 ± 0.0004	0.6887 ± 0.0003	0.6886 ± 0.0000	0.6886 ± 0.0000	0.6886 ± 0.0001	0.6886 ± 0.0001

Values are mean ± standard deviation across 100 simulated datasets. Low and high denote relative strata within the realistic-depth range. ANCOM-BC was excluded from this table because the exported phylum-level ANCOM-BC-related matrix was constant and therefore not valid for Shannon diversity testing.

Table S11: Shannon diversity index across preprocessing methods for the phylum-level realistic-depth unequal-depth positive scenario.

	Low-depth stratum		High-depth stratum		Full dataset	
**Method**	**Control**	**Case**	**Control**	**Case**	**Control**	**Case**
Raw data	0.9956 ± 0.0009	1.1141 ± 0.0005	0.9952 ± 0.0007	1.1139 ± 0.0004	0.9954 ± 0.0004	1.1140 ± 0.0003
TSS	0.9949 ± 0.0008	1.1136 ± 0.0005	0.9949 ± 0.0007	1.1136 ± 0.0004	0.9948 ± 0.0004	1.1136 ± 0.0003
CLR	0.9955 ± 0.0009	1.1138 ± 0.0005	0.9952 ± 0.0007	1.1137 ± 0.0004	0.9953 ± 0.0004	1.1137 ± 0.0003
CLR-BMR	0.9949 ± 0.0008	1.1136 ± 0.0005	0.9949 ± 0.0007	1.1136 ± 0.0004	0.9948 ± 0.0004	1.1136 ± 0.0003
CSS	0.9949 ± 0.0008	1.1136 ± 0.0005	0.9949 ± 0.0007	1.1136 ± 0.0004	0.9948 ± 0.0004	1.1136 ± 0.0003
DESeq2	0.9949 ± 0.0008	1.1136 ± 0.0005	0.9949 ± 0.0007	1.1136 ± 0.0004	0.9948 ± 0.0004	1.1136 ± 0.0003
edgeR-TMM	0.9949 ± 0.0008	1.1136 ± 0.0005	0.9949 ± 0.0007	1.1136 ± 0.0004	0.9948 ± 0.0004	1.1136 ± 0.0003
Rarefaction	0.9949 ± 0.0010	1.1134 ± 0.0009	0.9947 ± 0.0011	1.1135 ± 0.0010	0.9947 ± 0.0006	1.1134 ± 0.0006
ALDEx2	0.6892 ± 0.0007	0.8059 ± 0.0024	0.6886 ± 0.0001	0.8060 ± 0.0014	0.6888 ± 0.0003	0.8060 ± 0.0012

Values are mean ± standard deviation across 100 simulated datasets. Low and high denote relative strata within the realistic-depth range. ANCOM-BC was excluded from this table because the exported phylum-level ANCOM-BC-related matrix was constant and therefore not valid for Shannon diversity testing.

Table S12: Shannon diversity index across preprocessing methods for the phylum-level realistic-depth unequal-depth negative scenario.

	Low-depth stratum		High-depth stratum		Full dataset	
**Method**	**Control**	**Case**	**Control**	**Case**	**Control**	**Case**
Raw data	0.9956 ± 0.0009	0.9955 ± 0.0005	0.9953 ± 0.0006	0.9952 ± 0.0003	0.9954 ± 0.0004	0.9953 ± 0.0002
TSS	0.9949 ± 0.0009	0.9950 ± 0.0005	0.9949 ± 0.0006	0.9949 ± 0.0003	0.9949 ± 0.0004	0.9949 ± 0.0002
CLR	0.9956 ± 0.0009	0.9952 ± 0.0005	0.9952 ± 0.0006	0.9950 ± 0.0003	0.9953 ± 0.0004	0.9951 ± 0.0002
CLR-BMR	0.9949 ± 0.0009	0.9950 ± 0.0005	0.9949 ± 0.0006	0.9949 ± 0.0003	0.9949 ± 0.0004	0.9949 ± 0.0002
CSS	0.9949 ± 0.0009	0.9950 ± 0.0005	0.9949 ± 0.0006	0.9949 ± 0.0003	0.9949 ± 0.0004	0.9949 ± 0.0002
DESeq2	0.9949 ± 0.0009	0.9950 ± 0.0005	0.9949 ± 0.0006	0.9949 ± 0.0003	0.9949 ± 0.0004	0.9949 ± 0.0002
edgeR-TMM	0.9949 ± 0.0009	0.9950 ± 0.0005	0.9949 ± 0.0006	0.9949 ± 0.0003	0.9949 ± 0.0004	0.9949 ± 0.0002
Rarefaction	0.9949 ± 0.0010	0.9949 ± 0.0010	0.9948 ± 0.0011	0.9948 ± 0.0010	0.9948 ± 0.0006	0.9949 ± 0.0006
ALDEx2	0.6892 ± 0.0007	0.6886 ± 0.0000	0.6886 ± 0.0001	0.6886 ± 0.0000	0.6888 ± 0.0002	0.6886 ± 0.0000

Values are mean ± standard deviation across 100 simulated datasets. Low and high denote relative strata within the realistic-depth range. ANCOM-BC was excluded from this table because the exported phylum-level ANCOM-BC-related matrix was constant and therefore not valid for Shannon diversity testing.

Table S13: Number of significant Kruskal–Wallis tests (out of 100 repetitions) across preprocessing methods for the phylum-level Shannon diversity analysis under four realistic-depth simulation scenarios.

	Positive			Unequal-depth Positive			Negative			Unequal-depth Negative		
**Method**	**Low**	**High**	**Full**	**Low**	**High**	**Full**	**Low**	**High**	**Full**	**Low**	**High**	**Full**
Raw data	100	100	100	100	100	100	6	5	8	4	7	5
TSS	100	100	100	100	100	100	6	5	6	4	6	5
CLR	100	100	100	100	100	100	6	5	6	5	6	7
CLR-BMR	100	100	100	100	100	100	6	5	6	4	6	5
CSS	100	100	100	100	100	100	6	5	6	4	6	5
DESeq2	100	100	100	100	100	100	6	5	6	4	6	5
edgeR-TMM	100	100	100	100	100	100	6	5	6	4	6	5
Rarefaction	100	100	100	100	100	100	7	4	3	3	3	4
ALDEx2	100	100	100	100	100	100	1	6	3	6	6	8

Entries indicate the number of significant Kruskal–Wallis tests at $\alpha = 0.05$ across 100 simulated datasets. Positive scenarios assess recovery of the simulated diversity contrast, whereas negative scenarios assess Type I error behaviour. ANCOM-BC was excluded from this table because the exported phylum-level ANCOM-BC-related matrix was constant and therefore not valid for Shannon diversity testing.

1.2.2 Genus-level simulation

At genus level, the positive scenarios were again detected consistently, but the direction of the diversity contrast was reversed relative to the phylum simulation, with higher Shannon diversity in the control group (Tables S14 and S16). The balanced negative scenario remained close to the nominal false-positive range (Table S15). The main instability appeared in the unequal-depth negative scenario: CLR produced inflated Kruskal–Wallis significance counts, especially in the full dataset, whereas CLR-BMR was much closer to TSS, CSS, DESeq2, edgeR-TMM, and rarefaction (Tables S17 and S18). This indicates that the apparent CLR instability at genus level was partly driven by the pseudocount approach rather than by the CLR transformation alone.

Table S14: Shannon diversity index across preprocessing methods for the genus-level realistic-depth positive scenario.

	Low-depth stratum		High-depth stratum		Full dataset	
**Method**	**Control**	**Case**	**Control**	**Case**	**Control**	**Case**
Raw data	2.6300 ± 0.0013	2.3629 ± 0.0012	2.6301 ± 0.0006	2.3631 ± 0.0007	2.6301 ± 0.0006	2.3630 ± 0.0005
TSS	2.6296 ± 0.0013	2.3622 ± 0.0012	2.6300 ± 0.0006	2.3629 ± 0.0007	2.6299 ± 0.0006	2.3626 ± 0.0005
CLR	2.6331 ± 0.0014	2.3675 ± 0.0013	2.6310 ± 0.0006	2.3643 ± 0.0007	2.6318 ± 0.0006	2.3655 ± 0.0005
CLR-BMR	2.4302 ± 0.0704	2.3153 ± 0.0468	2.4305 ± 0.0706	2.3158 ± 0.0467	2.4304 ± 0.0705	2.3156 ± 0.0468
CSS	2.6296 ± 0.0013	2.3622 ± 0.0012	2.6300 ± 0.0006	2.3629 ± 0.0007	2.6299 ± 0.0006	2.3626 ± 0.0005
DESeq2	2.6296 ± 0.0013	2.3622 ± 0.0012	2.6300 ± 0.0006	2.3629 ± 0.0007	2.6299 ± 0.0006	2.3626 ± 0.0005
edgeR-TMM	2.6296 ± 0.0013	2.3622 ± 0.0012	2.6300 ± 0.0006	2.3629 ± 0.0007	2.6299 ± 0.0006	2.3626 ± 0.0005
Rarefaction	2.6289 ± 0.0018	2.3612 ± 0.0016	2.6286 ± 0.0018	2.3614 ± 0.0018	2.6287 ± 0.0011	2.3613 ± 0.0011
ALDEx2	2.6785 ± 0.0282	2.7730 ± 0.0220	2.6774 ± 0.0325	2.8072 ± 0.0239	2.6777 ± 0.0305	2.7911 ± 0.0232
ANCOM-BC	2.7163 ± 0.0095	2.3778 ± 0.0141	2.7326 ± 0.0081	2.4625 ± 0.0129	2.7254 ± 0.0087	2.4251 ± 0.0129

Values are mean ± standard deviation across 100 simulated datasets. Low and high denote relative strata within the realistic-depth range.

Table S15: Shannon diversity index across preprocessing methods for the genus-level realistic-depth negative scenario.

	Low-depth stratum		High-depth stratum		Full dataset	
**Method**	**Control**	**Case**	**Control**	**Case**	**Control**	**Case**
Raw data	2.6297 ± 0.0013	2.6302 ± 0.0011	2.6302 ± 0.0006	2.6303 ± 0.0007	2.6300 ± 0.0005	2.6303 ± 0.0005
TSS	2.6293 ± 0.0013	2.6295 ± 0.0011	2.6301 ± 0.0006	2.6301 ± 0.0007	2.6298 ± 0.0005	2.6299 ± 0.0005
CLR	2.6329 ± 0.0013	2.6331 ± 0.0011	2.6310 ± 0.0006	2.6310 ± 0.0007	2.6318 ± 0.0006	2.6319 ± 0.0005
CLR-BMR	2.4278 ± 0.0676	2.4279 ± 0.0676	2.4283 ± 0.0676	2.4283 ± 0.0676	2.4281 ± 0.0676	2.4282 ± 0.0676
CSS	2.6293 ± 0.0013	2.6295 ± 0.0011	2.6301 ± 0.0006	2.6301 ± 0.0007	2.6298 ± 0.0005	2.6299 ± 0.0005
DESeq2	2.6293 ± 0.0013	2.6295 ± 0.0011	2.6301 ± 0.0006	2.6301 ± 0.0007	2.6298 ± 0.0005	2.6299 ± 0.0005
edgeR-TMM	2.6293 ± 0.0013	2.6295 ± 0.0011	2.6301 ± 0.0006	2.6301 ± 0.0007	2.6298 ± 0.0005	2.6299 ± 0.0005
Rarefaction	2.6285 ± 0.0018	2.6288 ± 0.0016	2.6286 ± 0.0018	2.6289 ± 0.0015	2.6286 ± 0.0011	2.6288 ± 0.0010
ALDEx2	2.6776 ± 0.0271	2.6777 ± 0.0271	2.6764 ± 0.0312	2.6762 ± 0.0313	2.6768 ± 0.0293	2.6767 ± 0.0293
ANCOM-BC	2.7168 ± 0.0089	2.7170 ± 0.0088	2.7332 ± 0.0076	2.7331 ± 0.0080	2.7261 ± 0.0082	2.7260 ± 0.0082

Values are mean ± standard deviation across 100 simulated datasets. Low and high denote relative strata within the realistic-depth range.

Table S16: Shannon diversity index across preprocessing methods for the genus-level realistic-depth unequal-depth positive scenario.

	Low-depth stratum		High-depth stratum		Full dataset	
**Method**	**Control**	**Case**	**Control**	**Case**	**Control**	**Case**
Raw data	2.6292 ± 0.0016	2.3630 ± 0.0009	2.6301 ± 0.0009	2.3631 ± 0.0006	2.6296 ± 0.0008	2.3630 ± 0.0005
TSS	2.6286 ± 0.0016	2.3625 ± 0.0009	2.6298 ± 0.0009	2.3628 ± 0.0006	2.6292 ± 0.0008	2.3627 ± 0.0005
CLR	2.6344 ± 0.0015	2.3655 ± 0.0009	2.6327 ± 0.0009	2.3643 ± 0.0006	2.6334 ± 0.0008	2.3649 ± 0.0005
CLR-BMR	2.4094 ± 0.0318	2.3003 ± 0.0208	2.4102 ± 0.0315	2.3005 ± 0.0211	2.4098 ± 0.0317	2.3005 ± 0.0210
CSS	2.6286 ± 0.0016	2.3625 ± 0.0009	2.6298 ± 0.0009	2.3628 ± 0.0006	2.6292 ± 0.0008	2.3627 ± 0.0005
DESeq2	2.6286 ± 0.0016	2.3625 ± 0.0009	2.6298 ± 0.0009	2.3628 ± 0.0006	2.6292 ± 0.0008	2.3627 ± 0.0005
edgeR-TMM	2.6286 ± 0.0016	2.3625 ± 0.0009	2.6298 ± 0.0009	2.3628 ± 0.0006	2.6292 ± 0.0008	2.3627 ± 0.0005
Rarefaction	2.6282 ± 0.0017	2.3616 ± 0.0018	2.6286 ± 0.0016	2.3611 ± 0.0018	2.6284 ± 0.0010	2.3613 ± 0.0011
ALDEx2	2.6726 ± 0.0117	2.7778 ± 0.0105	2.6696 ± 0.0129	2.7984 ± 0.0107	2.6709 ± 0.0124	2.7885 ± 0.0106
ANCOM-BC	2.7112 ± 0.0088	2.4412 ± 0.0232	2.7218 ± 0.0083	2.4835 ± 0.0217	2.7169 ± 0.0083	2.4636 ± 0.0224

Values are mean ± standard deviation across 100 simulated datasets. Low and high denote relative strata within the realistic-depth range.

Table S17: Shannon diversity index across preprocessing methods for the genus-level realistic-depth unequal-depth negative scenario.

	Low-depth stratum		High-depth stratum		Full dataset	
**Method**	**Control**	**Case**	**Control**	**Case**	**Control**	**Case**
Raw data	2.6297 ± 0.0015	2.6303 ± 0.0009	2.6298 ± 0.0010	2.6302 ± 0.0007	2.6297 ± 0.0008	2.6303 ± 0.0004
TSS	2.6291 ± 0.0015	2.6299 ± 0.0009	2.6294 ± 0.0010	2.6300 ± 0.0007	2.6293 ± 0.0008	2.6299 ± 0.0004
CLR	2.6350 ± 0.0015	2.6319 ± 0.0009	2.6324 ± 0.0010	2.6310 ± 0.0007	2.6335 ± 0.0008	2.6314 ± 0.0004
CLR-BMR	2.4187 ± 0.0536	2.4192 ± 0.0535	2.4189 ± 0.0534	2.4193 ± 0.0535	2.4188 ± 0.0535	2.4193 ± 0.0535
CSS	2.6291 ± 0.0015	2.6299 ± 0.0009	2.6294 ± 0.0010	2.6300 ± 0.0007	2.6293 ± 0.0008	2.6299 ± 0.0004
DESeq2	2.6291 ± 0.0015	2.6299 ± 0.0009	2.6294 ± 0.0010	2.6300 ± 0.0007	2.6293 ± 0.0008	2.6299 ± 0.0004
edgeR-TMM	2.6291 ± 0.0015	2.6299 ± 0.0009	2.6294 ± 0.0010	2.6300 ± 0.0007	2.6293 ± 0.0008	2.6299 ± 0.0004
Rarefaction	2.6289 ± 0.0017	2.6284 ± 0.0019	2.6284 ± 0.0019	2.6284 ± 0.0018	2.6287 ± 0.0011	2.6284 ± 0.0009
ALDEx2	2.6758 ± 0.0200	2.6727 ± 0.0229	2.6733 ± 0.0216	2.6720 ± 0.0244	2.6745 ± 0.0208	2.6724 ± 0.0237
ANCOM-BC	2.7121 ± 0.0078	2.7133 ± 0.0075	2.7227 ± 0.0073	2.7223 ± 0.0069	2.7179 ± 0.0073	2.7181 ± 0.0071

Values are mean ± standard deviation across 100 simulated datasets. Low and high denote relative strata within the realistic-depth range.

Table S18: Number of significant Kruskal–Wallis tests (out of 100 repetitions) across preprocessing methods for the genus-level Shannon diversity analysis under four simulation scenarios.

	Positive			Unequal-depth Positive			Negative			Unequal-depth Negative		
**Method**	**Low**	**High**	**Full**	**Low**	**High**	**Full**	**Low**	**High**	**Full**	**Low**	**High**	**Full**
Raw data	100	100	100	100	100	100	3	6	8	7	6	10
TSS	100	100	100	100	100	100	3	5	5	9	10	13
CLR	100	100	100	100	100	100	3	5	5	42	18	65
CLR-BMR	100	100	100	100	100	100	3	5	6	9	10	12
CSS	100	100	100	100	100	100	3	5	5	9	10	13
DESeq2	100	100	100	100	100	100	3	5	5	9	10	13
edgeR-TMM	100	100	100	100	100	100	3	5	5	9	10	13
Rarefaction	100	100	100	100	100	100	5	4	7	2	9	5
ALDEx2	100	100	100	100	100	100	2	2	6	22	15	38
ANCOM-BC	100	100	100	100	100	100	1	4	2	4	5	0

Entries indicate the number of significant Kruskal–Wallis tests at $\alpha = 0.05$ across 100 simulated datasets. The positive and unequal-depth-positive scenarios assess detection performance, where values near 100 indicate consistent recovery of the simulated diversity difference and lower values imply a higher Type II error risk. The negative and unequal-depth-negative scenarios assess Type I error control, where values near 5 are expected by chance alone under nominal error control.

2 Comparison of normalization methods using multivariate analysis

2.1 Analysis of baseline-depth simulation scenarios

Following the error calculation from comparing oMEDA and the ground truth for 100 independent repetitions, an ANOVA model was applied to each positive scenario to identify statistical differences in errors between normalization methods. These ANOVA models incorporate three factors: the normalization method (including no normalization), the depth stratum (low-depth samples, high-depth samples and the complete dataset), and the repetition (the 100 individual repetitions). As detailed in Tables S19 through S22, the results consistently demonstrate that the normalization method is a statistically significant factor, accounting for over 92% of the variability in error values. This underscores that the choice of normalization is the most critical factor in accurately distinguishing prevalent taxa between control and case groups.

While the significance of the depth stratum and repetition varied by scenario and taxonomic resolution, their importance in terms of mean squares remained marginal compared to the normalization method, with the latter being many times more relevant in terms of mean squares.

Table S19: ANOVA results for the comparison of the estimated error across methods for the phylum positive scenario. Three factors were considered: the normalization method, the depth stratum (low-depth samples, high-depth samples, and the full dataset), and the repetition (the 100 simulated datasets). The table columns report sum of squares (SS), percentage of total sum of squares (%SS), degrees of freedom (df), mean squares (MS), F-statistic values (F), and p-values.

Source of variability	SS	%SS	df	MS	F	p-value
Normalization method	364.43	92.85	9	40.49	4589.42	< 0.001
Depth stratum	0.09	0.02	2	0.04	5.08	0.006
Repetition	2.46	0.63	99	0.02	2.82	< 0.001
Residuals	25.49	6.49	2889	0.01	—	—
Total	392.47	100.00	2999	—	—	—

Table S20: ANOVA results for the comparison of the estimated error across methods for the phylum unequal-depth scenario simulation. Three factors were considered: the normalization method, the depth stratum (low-depth samples, high-depth samples, and the full dataset), and the repetition (the 100 simulated datasets). The table columns report sum of squares (SS), percentage of total sum of squares (%SS), degrees of freedom (df), mean squares (MS), F-statistic values (F), and p-values.

Source of variability	SS	%SS	df	MS	F	p-value
Normalization method	914.60	98.49	9	101.62	23568.01	< 0.001
Depth stratum	0.27	0.03	2	0.13	30.91	< 0.001
Repetition	1.30	0.14	99	0.01	3.04	< 0.001
Residuals	12.46	1.34	2889	0.00	—	—
Total	928.62	100.00	2999	—	—	—

Table S21: ANOVA results for the comparison of the estimated error across methods for the genus positive scenario simulation. Three factors were considered: the normalization method, the depth stratum (low-depth samples, high-depth samples, and the full dataset), and the repetition (the 100 simulated datasets). The table columns report sum of squares (SS), percentage of total sum of squares (%SS), degrees of freedom (df), mean squares (MS), F-statistic values (F), and p-values.

Source of variability	SS	%SS	df	MS	F	p-value
Normalization method	78.01	95.84	9	8.67	9256.38	< 0.001
Depth stratum	0.47	0.57	2	0.23	248.55	< 0.001
Repetition	0.22	0.27	99	0.00	2.35	< 0.001
Residuals	2.71	3.32	2889	0.00	—	—
Total	81.40	100.00	2999	—	—	—

Table S22: ANOVA results for the comparison of the estimated error across methods for the genus unequal-depth scenario simulation. Three factors were considered: the normalization method, the depth stratum (low-depth samples, high-depth samples, and the full dataset), and the repetition (the 100 simulated datasets). The table columns report sum of squares (SS), percentage of total sum of squares (%SS), degrees of freedom (df), mean squares (MS), F-statistic values (F), and p-values.

Source of variability	SS	%SS	df	MS	F	p-value
Normalization method	493.20	98.47	9	54.80	21802.00	< 0.001
Depth stratum	0.11	0.02	2	0.05	21.39	< 0.001
Repetition	0.29	0.06	99	0.00	1.15	0.15
Residuals	7.26	1.45	2889	0.00	—	—
Total	500.85	100.00	2999	—	—	—

2.2 Analysis of realistic-depth simulation scenarios

This section explores four additional scenarios designed to mimic the realistic sequencing depths typically found in metagenomics. These scenarios share the exact same characteristics and ground-truth biological compositions as those discussed in the main text; they differ only in their total number of reads.

The four scenarios vary based on two factors: whether a true biological difference exists between the groups (positive vs. negative), and whether the sequencing depth is balanced between the groups. In both equal-depth scenarios, sequencing depth varies from 10,000 to 100,000. In contrast, in the unequal-depth scenarios, control samples present 10,000 to 30,000 reads, while case samples exhibit 30,000 to 90,000 reads.

Tables S23 and S24 display the average error values comparing oMEDA results to the ground truth. Consistent with scenarios featuring depth ranges in the hundreds of reads, edgeR-TMM remains the best-performing method overall.

For the phylum-level results (Table S23), edgeR-TMM is the optimal normalization method, consistently securing the lowest estimated error. Standard scaling methods like Rarefaction and TSS provide stable but moderately higher errors, and CSS performs poorly with the highest errors. Log-ratio methods like CLR and CLR-MR, ALDEx2 and ANCOM-BC behave similarly to each other. Under the positive scenario, omitting normalization yields a moderate error, but this value spikes in the unequal-depth positive scenario, again underscoring how vital normalization becomes when sample sequencing depths are highly unbalanced.

At the genus-level (Table S24), the results favor both DESeq2 and edgeR-TMM as the optimal methods. In contrast to the phylum level, log-ratio approaches undergo severe performance degradation at the genus level. Notably, CLR performs significantly worse than CLR-MR. In general, log-ratio methods yield significantly higher errors than simple

Table S23: Estimated error after applying normalization methods (mean ± std) for the phylum positive and unequal-depth positive realistic-depth scenarios, with sequencing depths on the order of tens of thousands of reads per sample.

	Positive			Unequal-depth Positive		
**Normalization method**	**Low**	**High**	**Full**	**Low**	**High**	**Full**
Raw data	0.540 ± 0.413^e	0.250 ± 0.201^b	0.335 ± 0.279^c	1.853 ± 0.015^g	1.854 ± 0.008^g	1.853 ± 0.010^g
CLR	0.445 ± 0.007^d	0.445 ± 0.004^d	0.445 ± 0.003^d	0.444 ± 0.008^d	0.443 ± 0.005^d	0.443 ± 0.004^d
CLR-BMR	0.447 ± 0.007^d	0.445 ± 0.004^d	0.446 ± 0.003^d	0.447 ± 0.008^d	0.444 ± 0.006^d	0.446 ± 0.004^d
CSS	1.401 ± 0.003^g	1.400 ± 0.002^f	1.400 ± 0.002^f	1.401 ± 0.004^f	1.401 ± 0.003^f	1.401 ± 0.002^f
DESeq2	0.714 ± 0.013^f	0.714 ± 0.007^e	0.714 ± 0.006^e	0.714 ± 0.013^e	0.714 ± 0.010^e	0.714 ± 0.006^e
Rarefaction	0.230 ± 0.007^b	0.230 ± 0.007^b	0.230 ± 0.004^b	0.229 ± 0.007^b	0.230 ± 0.008^b	0.230 ± 0.004^b
TSS	0.231 ± 0.005^b	0.230 ± 0.003^b	0.230 ± 0.002^b	0.230 ± 0.005^b	0.230 ± 0.004^b	0.230 ± 0.002^b
edgeR-TMM	$\mathbf{0.010 \pm 0.009^a}$	$\mathbf{0.013 \pm 0.015^a}$	$\mathbf{0.011 \pm 0.012^a}$	$\mathbf{0.018 \pm 0.012^a}$	$\mathbf{0.024 \pm 0.016^a}$	$\mathbf{0.020 \pm 0.014^a}$
ALDEx2	0.446 ± 0.008^d	0.445 ± 0.004^d	0.446 ± 0.003^d	0.447 ± 0.008^d	0.444 ± 0.006^d	0.445 ± 0.004^d
ANCOM-BC	0.338 ± 0.005^c	0.337 ± 0.003^c	0.337 ± 0.002^c	0.336 ± 0.006^c	0.336 ± 0.004^c	0.336 ± 0.003^c

Entries are mean estimated error ± standard deviation across 100 simulated datasets. Methods sharing a letter within a column are not significantly different at the 0.01 level. The lowest mean value in each column is highlighted in bold. Abbreviations: CLR, centered log-ratio; CSS, cumulative sum scaling; TSS, total sum scaling; edgeR-TMM, trimmed mean of M-values normalization implemented in edgeR; DESeq2, median-ratio size-factor normalization implemented in DESeq2.

Table S24: Estimated error after applying normalization methods (mean ± std) for the genus positive and unequal-depth positive realistic-depth scenarios, with sequencing depths on the order of tens of thousands of reads per sample.

	Positive			Unequal-depth Positive		
**Normalization method**	**Low**	**High**	**Full**	**Low**	**High**	**Full**
Raw data	0.095 ± 0.092^c	0.077 ± 0.024^b	0.077 ± 0.037^b	1.359 ± 0.022^g	1.361 ± 0.010^h	1.361 ± 0.015^h
CLR	0.860 ± 0.019^f	1.071 ± 0.008^f	0.976 ± 0.010^e	0.947 ± 0.011^e	1.062 ± 0.007^f	1.007 ± 0.006^f
CLR-BMR	0.528 ± 0.018^d	0.524 ± 0.008^c	0.525 ± 0.007^c	0.534 ± 0.014^c	0.526 ± 0.009^c	0.529 ± 0.006^c
CSS	0.709 ± 0.018^e	0.731 ± 0.005^d	0.724 ± 0.006^d	0.727 ± 0.010^d	0.733 ± 0.006^d	0.730 ± 0.005^d
DESeq2	$\mathbf{0.003 \pm 0.001^a}$	$\mathbf{0.004 \pm 0.000^a}$	$\mathbf{0.004 \pm 0.000^a}$	$\mathbf{0.003 \pm 0.001^a}$	$\mathbf{0.004 \pm 0.001^a}$	$\mathbf{0.004 \pm 0.000^a}$
Rarefaction	0.077 ± 0.002^b	0.077 ± 0.002^b	0.077 ± 0.001^b	0.077 ± 0.002^b	0.077 ± 0.002^b	0.077 ± 0.001^b
TSS	0.077 ± 0.001^b	0.077 ± 0.001^b	0.077 ± 0.001^b	0.077 ± 0.001^b	0.077 ± 0.001^b	0.077 ± 0.001^b
edgeR-TMM	$\mathbf{0.005 \pm 0.003^a}$	$\mathbf{0.005 \pm 0.004^a}$	$\mathbf{0.005 \pm 0.003^a}$	$\mathbf{0.003 \pm 0.002^a}$	$\mathbf{0.002 \pm 0.002^a}$	$\mathbf{0.003 \pm 0.002^a}$
ALDEx2	1.092 ± 0.021^g	1.295 ± 0.007^g	1.208 ± 0.010^f	1.184 ± 0.011^f	1.287 ± 0.007^g	1.239 ± 0.006^g
ANCOM-BC	0.867 ± 0.017^f	1.061 ± 0.007^e	0.973 ± 0.010^e	0.945 ± 0.010^e	1.051 ± 0.007^e	1.000 ± 0.005^e

Entries are mean estimated error ± standard deviation across 100 simulated datasets. Methods sharing a letter within a column are not significantly different at the 0.01 level. The lowest mean value in each column is highlighted in bold. Abbreviations: CLR, centered log-ratio; CSS, cumulative sum scaling; TSS, total sum scaling; edgeR-TMM, trimmed mean of M-values normalization implemented in edgeR; DESeq2, median-ratio size-factor normalization implemented in DESeq2.

scaling techniques like TSS and Rarefaction, which remain highly stable. Furthermore, the unequal-depth positive scenario again induces a large error when utilizing raw data.

3 Supplementary PCA visualizations and discussion

This section contains Principal Component Analysis (PCA) representations of raw and normalized datasets that help visualize how normalization affects the separation (or lack thereof) between control and case groups and the variability driven by sequencing depth.

3.1 PCA visualizations for baseline-depth simulations

3.1.1 Negative scenario PCA score patterns

In the negative scenario, no true biological difference exists between control and case samples. As expected, Fig. S1 shows that, in the phylum-level negative scenario, control and case samples overlap extensively, with no clear group separation in the PC1–PC2 space accounting for 98% of total variability in raw data. This lack of discriminative structure indicates that such datasets should be screened before multivariate analysis with oMEDA and excluded when no meaningful between-group distinction is present. Because oMEDA will always find a numerical difference between groups, interpreting results without a real between-group distinction is misleading.

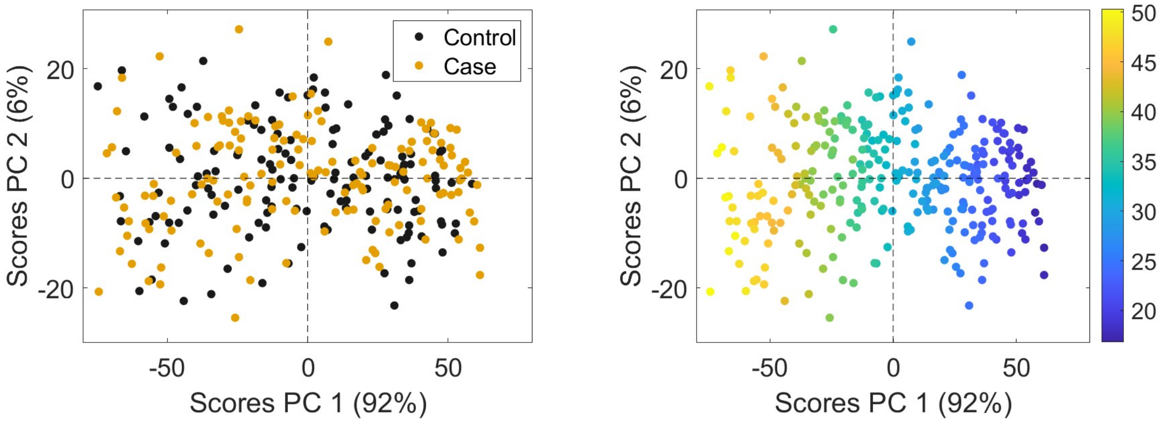

(a) Scores colored by group. **(b)** Scores colored by sequencing depth.

Figure S1: Phylum-level negative scenario PCA score plots for the raw data. Control and case samples show substantial overlap, indicating the absence of a clear biological separation. The depth-colored representation likewise does not reveal a class-related structure. In such case, datasets without meaningful group discrimination should be excluded from downstream multivariate interpretation.

Similarly, Fig. S2 shows that control and case samples exhibit complete overlap in the genus-level negative scenario, with no clear separation between groups in the score space.

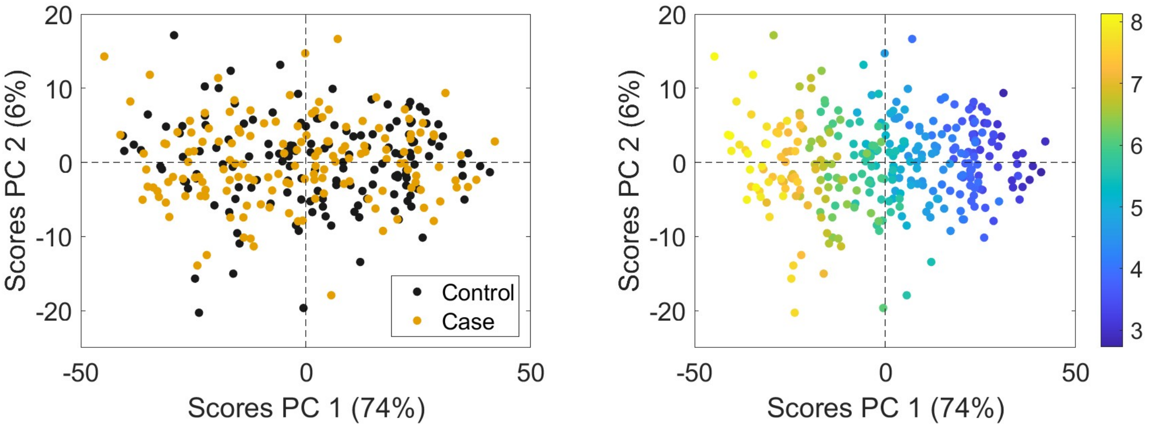

(a) Scores colored by group. **(b)** Scores colored by sequencing depth.

Figure S2: Genus-level negative scenario PCA score plots for the raw data. The group-colored score plot shows strong overlap between control and case samples, indicating no clear biological separation. The depth-colored score plot likewise does not reveal a distinct class-related structure. In such case, datasets without meaningful group discrimination should be excluded from downstream multivariate analysis.

3.1.2 PCA visualizations at phylum level

This section provides a detailed phylum-level comparison of PCA and oMEDA representations of one of the simulated datasets in the positive scenario across normalization methods. Fig. S3 shows, for each method, the PCA scores colored by group, the PCA scores colored by sequencing depth, the PCA loadings, and the corresponding oMEDA pattern, allowing direct visual comparison with the predefined ground-truth direction. Positive bars in the oMEDA plot indicate a higher count of the corresponding taxa in case samples, whereas negative bars indicate a higher count in control samples.

Starting with TSS, the score plot in Fig. S3(a) shows a clear but still moderate separation between control and case samples, indicating that the biological contrast is recovered but not maximally sharpened. The loading pattern in Fig. S3(c) is broadly consistent with the ground truth shown in Fig. 6(b) in the main document, with *Pseudomonadota* and *Bacteroidota* being associated with case samples, and *Bacillota* being more prominent in control samples. *Actinomycetota* remains secondary. The oMEDA profile in Fig. S3(d) follows the same overall direction, although its magnitude departs from the reference pattern. This agrees with the numerical results, where TSS gives relatively low error values (0.232–0.235 across strata), indicating good proximity to the ground truth, but without reaching the best-performing solution.

Rarefaction produces a similarly structured result. The class score plot in Fig. S3(e) shows visible group separation that is slightly more compact than in TSS, while the depth-colored score plot in Fig. S3(f) suggests that the primary organization remains bi-

ologically interpretable, as rarefaction makes depth uniform among samples. The loading configuration in Fig. S3(g) again preserves the main biological axis defined by the opposition between *Bacillota* and the case-associated taxa, especially *Pseudomonadota*, in agreement with the ground truth in Fig. 6(b) in the main document. Its oMEDA profile in Fig. S3(h) remains directionally concordant with the ground truth, although with some attenuation of smaller contributions. In terms of distance from the reference, rarefaction is nearly indistinguishable from TSS, with mean errors of 0.228–0.238, confirming that both methods recover the correct biological direction with comparable accuracy.

CLR shows stronger visual alignment with the expected biological structure. The group-colored score plot in Fig. S3(i) displays clearer group separation even when considering only one principal component, and the depth-colored score plot in Fig. S3(j) indicates that the dominant structure remains aligned with the biological contrast rather than with sequencing depth. The loading map in Fig. S3(k) preserves the same directional organization seen in the ground truth in Fig. 6(b) in the main document, with positive contributions from *Pseudomonadota* and *Bacteroidota* and a negative contribution from *Bacillota*. However, the relative balance among phyla is less faithful than in the best-performing methods, and the oMEDA pattern in Fig. S3(l) appears amplified for some taxa like *Pseudomonadota*, and very attenuated for *Bacillota*. This is reflected in the larger quantitative distance from the ground truth, with mean errors of 0.356–0.382. Thus, CLR improves visual separation, but this sharper ordination does not translate into closer agreement with the true reference structure.

In CSS, the group separation is mainly visible along the first component in Fig. S3(m), although the overlap between groups is larger than in the previous methods. The loading and oMEDA patterns reveal a poor fidelity to the reference. Although the loading map in Fig. S3(o) remains directionally interpretable and the dominant contrast is still biologically meaningful when compared with the ground truth in Fig. 6(b) in the main document, the relative contributions of taxa are heavily inflated. This distortion is even more evident in the oMEDA profile in Fig. S3(p), where the magnitudes deviate substantially from the expected reference pattern, with *Bacteroidota* being incorrectly deemed irrelevant in the difference between groups. This is fully consistent with the distance analysis, where CSS yields by far the largest error values (1.408–1.413), making it the furthest method from the ground truth.

DESeq2 provides weaker recovery of the target biological structure than TSS or rarefaction. The class score plot in Fig. S3(q) still shows some separation between control and case samples, but with greater spread and weaker cohesion than in the better-performing methods. The depth-colored score plot in Fig. S3(r) indicates that the structure is less stable overall. The correspondence between the loading configuration in Fig. S3(s) and the ground-truth direction in Fig. 6(b) in the main document is therefore less precise.

Likewise, the oMEDA profile in Fig. S3(t) retains the broad sign pattern of the major taxa, but the taxon-level balance is not recovered as faithfully as in TSS or rarefaction. Its mean errors (0.446–0.463) confirm that DESeq2 is more distant from the reference structure than TSS, rarefaction, or CLR.

Comparison against the ground truth in Fig. 6(b) in the main document shows that TSS and rarefaction are the closest among the methods displayed in Fig. S3, both preserving the expected biological direction with the smallest distances to the reference. CLR improves visual class separation, as seen in Fig. S3(i), but at the cost of a larger deviation from the true structure, evident in Fig. S3(l). DESeq2 performs more weakly, whereas CSS is the furthest from the ground truth and therefore the least faithful representation of the known simulated signal. These observations are consistent with the error summary, where rarefaction and TSS form the low-error group, CLR and DESeq2 show intermediate distortion, and CSS is significantly worse. For completeness, the best overall method in the full numerical comparison was edgeR-TMM, with the lowest mean error across all strata. PCA and oMEDA visualizations for edgeR-TMM are available in the main text.

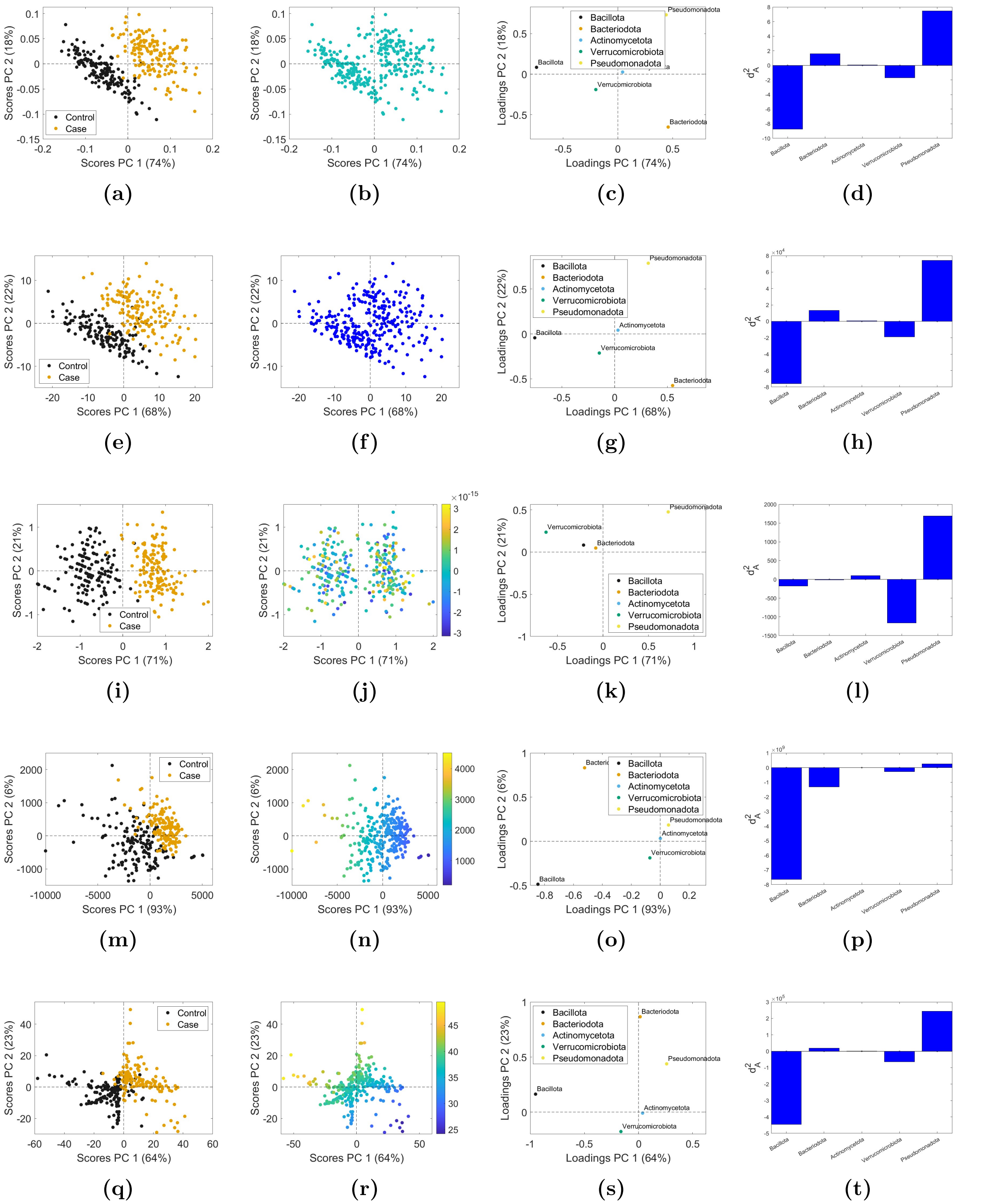

Figure S3: Phylum-level PCA and oMEDA representations across normalization methods. Panels (a)–(d) correspond to TSS and show, respectively, PCA scores colored by sample group, PCA scores colored by sequencing depth, PCA loadings, and the corresponding oMEDA plot. Panels (e)–(h) correspond to rarefaction and show the same sequence of representations. Panels (i)–(l) correspond to CLR, panels (m)–(p) to CSS, and panels (q)–(t) to DESeq2. In each four-panel set, the first panel shows group separation, the second shows the effect of sequencing depth, the third summarizes the contribution of individual phyla to the principal-component structure, and the fourth highlights the taxa driving the class contrast.

3.1.3 PCA visualizations at genus level

This section provides an overview of PCA visualizations of one of the genus-level simulated datasets in the positive scenario after applying normalization methods. The score and loading patterns are provided in Figs. S4–S8. Raw data often show the strongest natural separation between groups because the original abundance levels are preserved. Rarefaction can reduce this separation by discarding reads to equalize depth. Transformations such as CLR, TSS, and CSS adjust for compositional effects, but may compress differences between groups depending on the data structure. In contrast, count-based methods such as edgeR model abundance differences directly and may detect group effects statistically even when visual separation in transformed data appears weaker (see Fig. S8).

Across the depth-related datasets, rarefaction shows the clearest separation between the control and case groups (Fig. S4). CLR (Fig. S5) and TSS (Fig. S6) produce tight score ranges that can lead to overlap, while CSS (Fig. S7) captures little variation and separates groups poorly. edgeR (Fig. S8) shows high variance but is affected by strong scaling, making the visual separation less stable. The raw genus-level data also separate groups well, but remain influenced by depth differences. In contrast, rarefaction provides both high variance and stable scaling, making it the most effective method for visualizing group distance under depth variation.

When comparing PC1 loadings to the biological effect sizes, the raw genus-level data show the strongest overall agreement. Their high PC1 variance and wide loading range allow the major genera with large effect sizes, such as *Prevotella*, *Bacteroides*, and *Oscillospira*, to appear more prominently along PC1, making their influence easier to detect. CLR also performs reasonably well, maintaining good variance and preserving relative differences between taxa, which helps it capture part of the biological pattern even if the loadings are more compressed. In contrast, CSS explains less variation and therefore aligns more weakly with the true effect sizes. Methods that mainly provide feature ranking or reduced interpretability at the loading level, such as edgeR, rarefaction, and TSS, are less straightforward to compare quantitatively in this framework. Overall, the raw genus-level representation offers the closest match to the biological signals in the effect-size data. However, despite the perfect separation between groups along PC1 for all normalization methods, the recovery of taxa-level abundance differs across methods, as evidenced by the comparison of oMEDA and the ground truth performed in the main text.

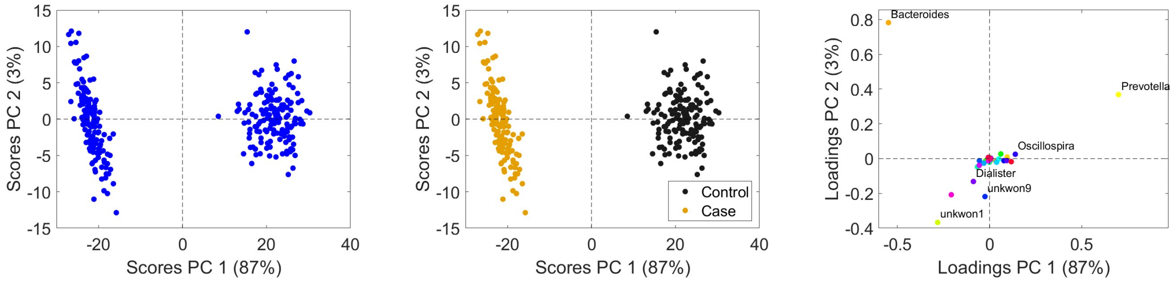

(a) (b) (c)

Figure S4: PCA visualization for Rarefaction normalization at the genus level. Each panel shows different aspects of the data: (a) colored by sequencing depth, (b) colored by the two ecosystems, and (c) genus-level loadings with 36 taxa.

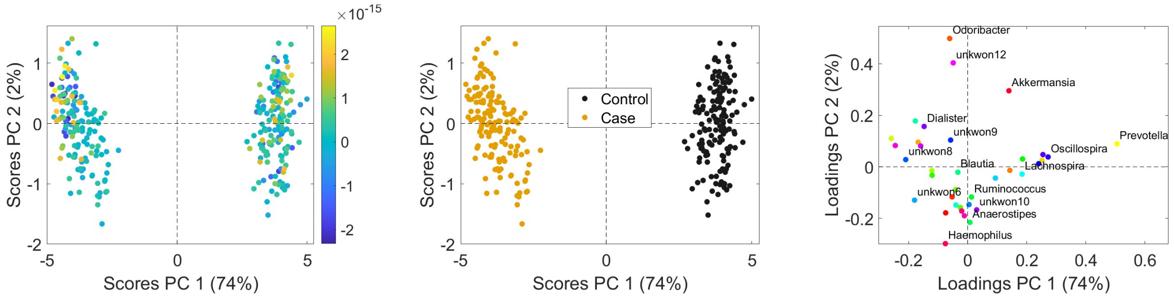

(a) (b) (c)

Figure S5: PCA visualization for Centered log-ratio (CLR) normalization at the genus level. Each panel shows different aspects of the data: (a) colored by sequencing depth, (b) colored by the two ecosystems, and (c) genus-level loadings with 36 taxa.

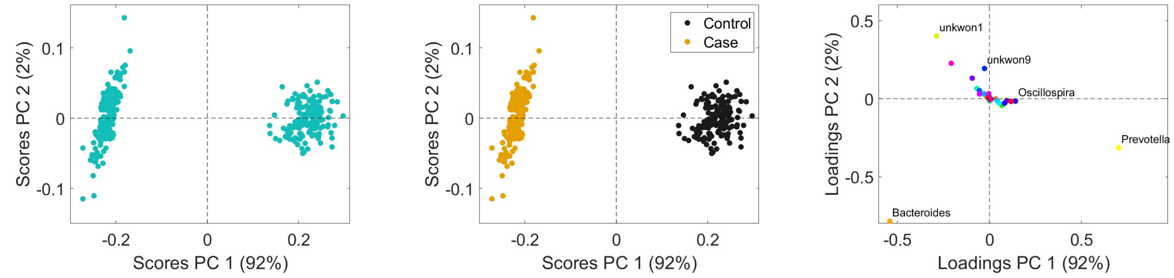

(a) (b) (c)

Figure S6: PCA visualization for Total Sum Scale (TSS) normalization at the genus level. Each panel shows different aspects of the data: (a) colored by sequencing depth, (b) colored by the two ecosystems, and (c) genus-level loadings with 36 taxa.

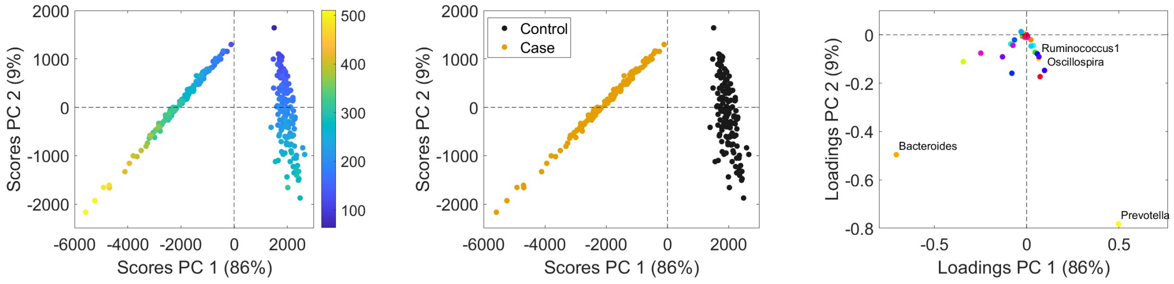

(a) (b) (c)

Figure S7: PCA visualization for Cumulative Sum Scaling (CSS) normalization at the genus level. Each panel shows different aspects of the data: (a) colored by sequencing depth, (b) colored by the two ecosystems, and (c) genus-level loadings with 36 taxa.

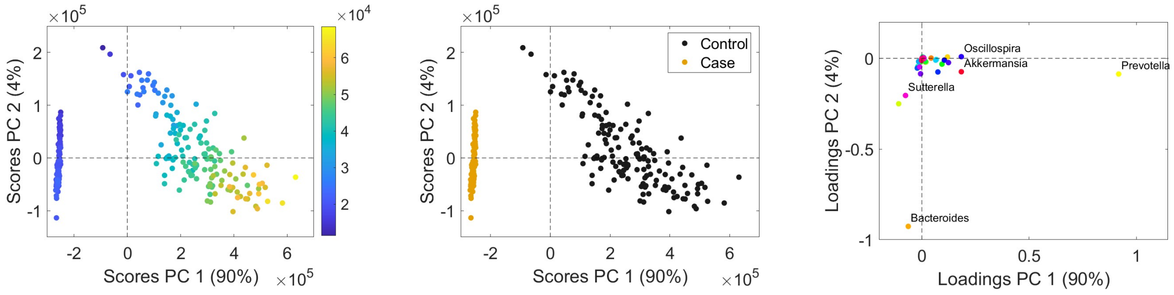

(a) (b) (c)

Figure S8: PCA visualization for edgeR normalization at the genus level. Each panel shows different aspects of the data: (a) colored by sequencing depth, (b) colored by the two ecosystems, and (c) genus-level loadings with 36 taxa.

3.1.4 PCA visualizations for realistic-depth simulations

For completeness, PCA visualizations of the raw data were generated under the realistic-depth positive scenario. These plots provide only a descriptive view of sample-level structure before normalization and should not be interpreted as evidence of normalization accuracy.

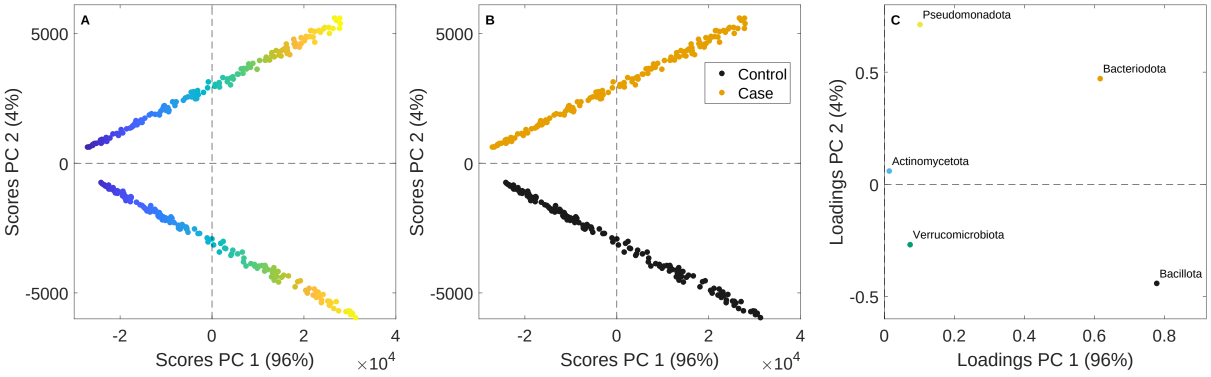

Figure S9: PCA visualization of the raw phylum-level data under the realistic-depth positive scenario. (A) Scores colored by sequencing depth. (B) Scores colored by group. (C) Phylum-level loadings.

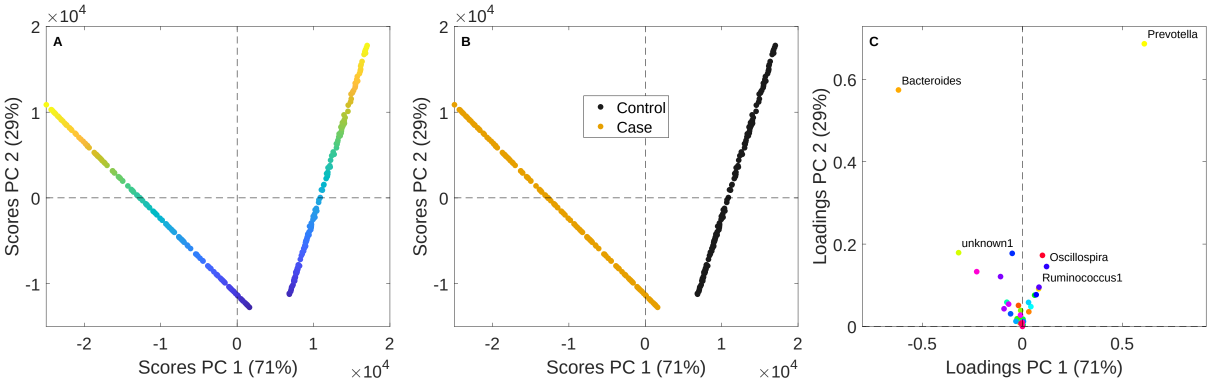

Figure S10: PCA visualization of the raw genus-level data under the realistic-depth positive scenario. (A) Scores colored by sequencing depth. (B) Scores colored by group. (C) Genus-level loadings.
